# In silico, in vitro, and in vivo evaluation of the antiviral potential of glycyrrhizic acid and chitosan nanoparticles against foot and mouth disease virus

**DOI:** 10.1186/s12985-026-03271-6

**Published:** 2026-08-14

**Authors:** Asmaa R.F. Eweis, Khodeir M. Hassan, Sahar A.H. Shoman, Hoda A. Taha, Ehab E. Ibrahim, Tahsin Shoala

**Affiliations:** 1 Microbiology Department, Faculty of Science, Ain Shams University, Cairo, Egypt; 2 Agriculture Research Center (ARC), Veterinary Serum and Vaccine Research Institute (VSVRI), Abasia, Cairo, Egypt; 3 College of Biotechnology, Misr University for Science and Technology, 6th of October City, Giza, Egypt

**Keywords:** FMD, GA-NPs, Ch-NPs, Antiviral

## Abstract

**Background:**

Foot-and-mouth disease (FMD) considers as one of the extremely infectious diseases with major economic impact on cloven-hoofed animals globally. The causative agent of the disease, Foot and Mouth Disease Virus (FMDV), comprises seven serotypes designated as; O, A, SAT1, SAT2, SAT3, C, and Asia1. Over the past decades, studies have shown that glycyrrhizic acid, the primary bioactive compound of licorice root, and chitosan, as a biodegradable and biocompatible natural polymer derived from crustacean shells, are potent candidates for inhibition of FMDV activity. The current work evaluated the potential antiviral efficacy of glycyrrhizic acid nanoparticles (GA-NPs) and chitosan nanoparticles (Ch-NPs) against different FMDV serotypes through computational (molecular docking), in vitro and in vivo assays.

**Materials and methods:**

The study included the use of field isolates from local FMD viral outbreaks (O PanAsia-2, A Iran 05, A Africa 2020, A Africa G IV Egypt 2022, A Venezuela, and SAT2/EGY/2012); Glycyrrhizic acid and chitosan nanoparticles were prepared and characterized; antiviral activity was assessed by molecular docking analysis, BHK-21 cell assays, and experimental animal studies using representative FMDV serotypes.

**Results:**

Molecular docking showed that GA-NPs exhibited stronger binding affinity toward the FMDV RNA-dependent RNA polymerase (3D^pol^) with binding energies of -8.974 kcal/mol while Ch-NPs demonstrated superior binding affinity toward the FMDV VP3 protein with binding energies of -7.173 kcal/mol, respectively. At a concentration of 2 × 10^− 1^ mg/ml, both nanoparticles significantly suppressed viral replication, yielding a reduction in viral titers 8, 7.5, 7, 7, 7.5, and 6.5 log₁₀ TCID₅₀/ml for O PanAsia-2, A Iran 05, A Africa 2020, A Africa G IV Egypt 2022, A Venezuela, and SAT2/EGY/2012, respectively which can aid in the disease restriction during outbreaks. Otherwise, at 2 × 10^− 2^ mg/ml, treatment markedly reduced viral dissemination in treated guinea pigs, providing protection rates of 66.7%; 44.4%; 66.7%; 100%; and 77.8% and 100% against serotype O; A Iran 05; A Africa 2020; A Africa G IV Egypt 2022; A Venezuela; and SAT2, compared with untreated controls.

**Conclusion:**

These findings highlighted that GA-NPs and Ch-NPs possess broad-spectrum antiviral activity against six FMDV serotypes, supporting their further development as nanoparticle-based antiviral agents for the control of FMD.

## Background

FMD as one of the most pervasive diseases impacting both domestic and wild cloven-hoofed animals with adverse consequences on meat and milk production, as opposed to convalescent animals becoming carriers and source of infection [1]. It is a highly transmittable viral disease between universal livestock in the terms of economic impact and interfering the animals’ trade on local and international levels and people movement constraint which influence the tourism sector [2]. FMD significantly influence the economy owing to its capability to cause losses of production, decreased milk yield, prenatal mortalities, premature culling, abortion, local and international constraining of animals’ trade accomplished with movement limitation of people according to [3]. The causal agent of FMD is a member of the family picornaviridae of the genus aphthovirus, possessing a small positive sense RNA genome around a size of 8.5 kb [4]. FMDV can be occurred in diverse serotypes involving A, O, SAT1, SAT2, SAT3, C and Asia 1 with great number of subtypes belongs to each serotype [5].

Throughout 2021–2022, ten Egyptian provinces were affected by several FMDV strains (O, A, SAT2) that were identified in buffaloes and cattle by performing ELISA. The distinct symptoms exhibited in cattle are fever, drooling, lip smacking, shivering and lesions on dental pad, in interdigital cleft, on tongue and along the coronary band of the hoof. These signs appear 24–48 h after infection. It was showed that the virus causes an extremely contagious disease in wild ruminants and pigs [6].

With the aim of creating modern materials, structures, and devices at the supramolecular plane, nanotechnology began to evolve in the mid-20^th^ century, where nanoparticles are quite small finite-sized particles extending from 1 to 100 nanometers (nm; 1 nm = 10⁻⁹ meters). Due to their nanoscale size, nanoparticles manifest unique physical and chemical traits, making them invaluable in plentiful fields. They have various applications, including in medicine, particularly profitable for improving medical imaging of organs and tumors, facilitating targeted drug delivery, and supporting tissue and bone regeneration [7].

Glycyrrhizic acid (GA), also called glycyrrhizin, and glycyrrhetinic acid (GA), which are extracted from the dried roots of the licorice shrub, serve as potent antioxidant, anti-inflammatory, antiviral, antitumor agents, and immunoregulators [8]. Chitosan which is derived from chitin, the constituent of the fungal cell wall and the shells of insects. It was reported that chitosan-based anti-coronavirus compounds under in vitro and in vivo conditions can suppress the infection efficiently [9]. Nevertheless, both are slowly soaked up in vivo because of its reduced aqueous dissolution. Extensive studies have indicated that the decline of the size of drug particle to the nanoscale significantly elevates the efficacy and bioavailability of the drug.

The current investigation was developed to examine the effect of GA-NPs and Ch-NPs as prospective candidate antiviral agents against FMDV.

## Materials and methods

*Ethical approval:

This work was confirmed by the Animal Ethics Committee of the Veterinary Serum and Vaccine Research Institute (VSVRI), and Research Ethics Committee of Ain Shams University, Microbiology Department with number: ASU-SCI/MICR/2024/5/4. All experiments matched with the VSVRI guidelines for animal research.

## In silico study

### Molecular docking

#### Selection and preparation of ligands

The chemical structures of chitosan and glycyrrhizic acid were procured from PubChem database (http://pubchem.ncbi.nlm.nih.gov), as clarified in Table (1), with (PubChem CID: 14982) for glycyrrhizic acid and (PubChem CID: 129662530) for chitosan as FMDV inhibitors candidates. These ligands were downloaded as 3D-structurte data file (SDF) format then underwent energy minimization using Chem3D 16.0 and geometrical optimization via gaussian 16 programs. The final optimized structures were converted into PDB format [10].

**Fig. 1:**
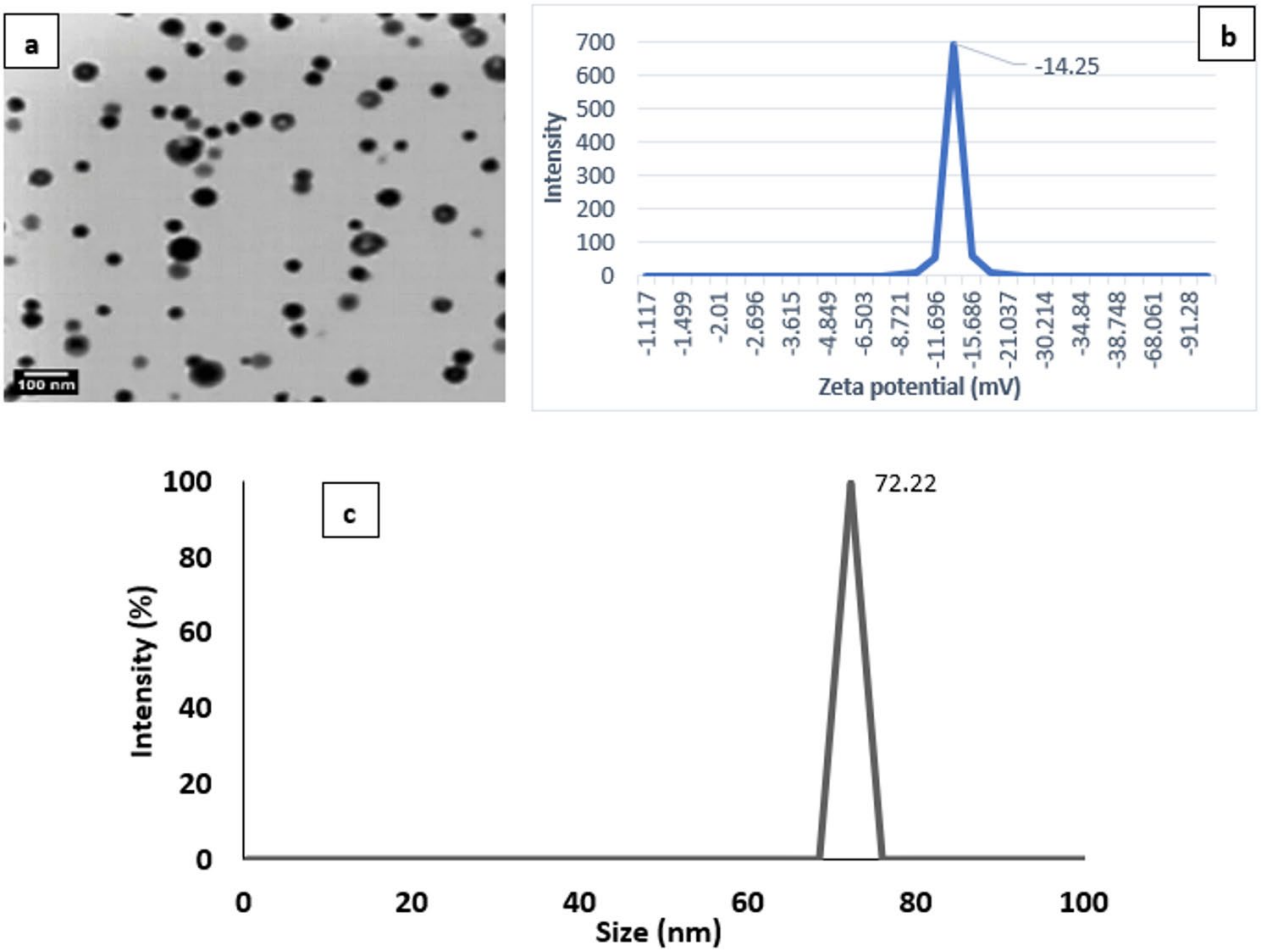

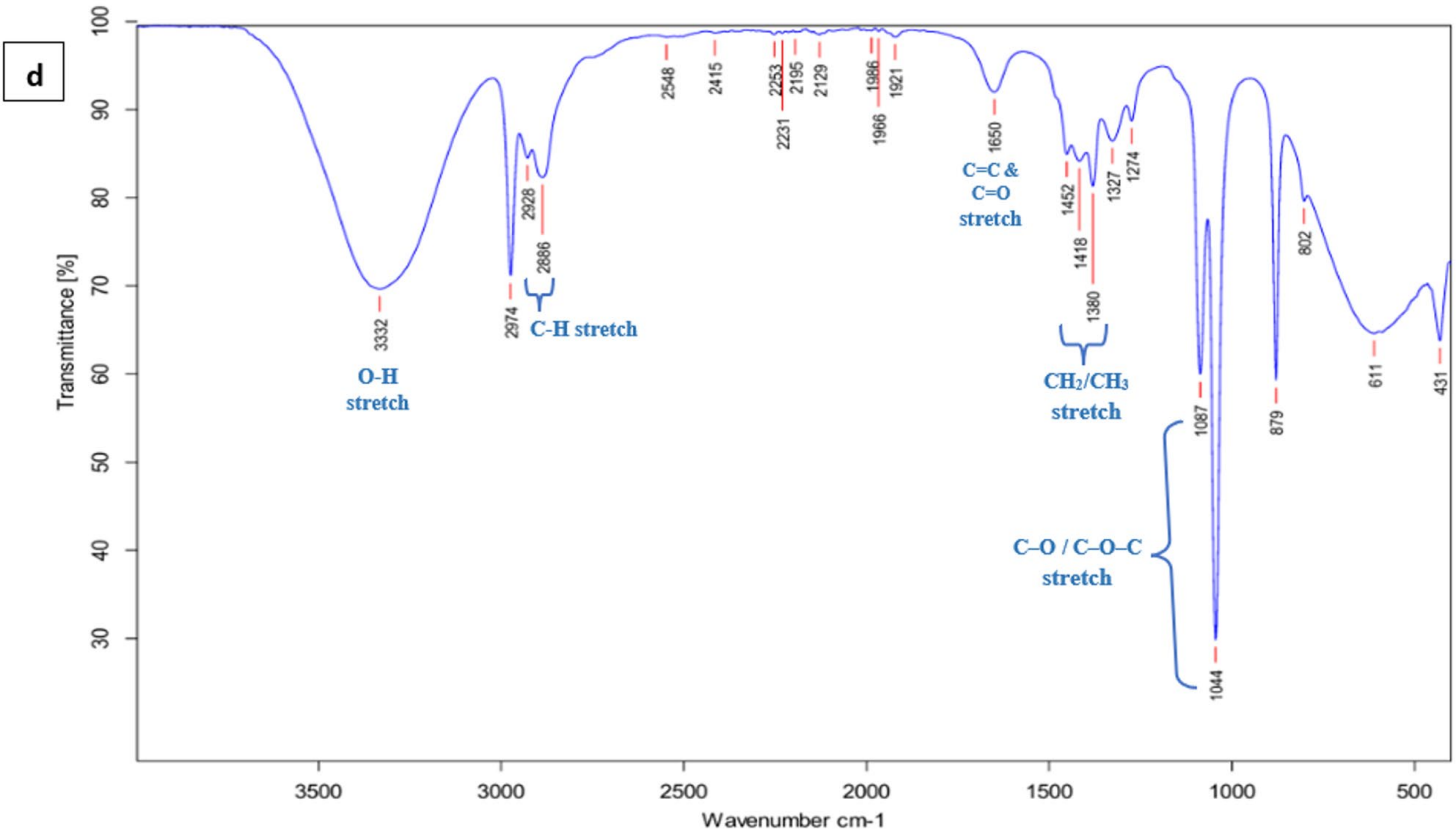
Characterization of GA-NPs. **a: **TEM of GA-NPs **b:** Zeta potential of GA-NPs **c:** DLS of GA-NPs **d:** FTIR spectrum of GA-NPs

#### Selection and preparation of target proteins

The 3D structures of six FMDV proteins were retrieved from RCSB Protein Data Bank (RCSB PDB) (http://www.rcsb.org). Each were identified with specific PDB ID such as (VP1, VP2, VP3 and VP4) complex (PDB ID: 7DSS), 3C protease (3C^pro^) (PDB ID: 2WV4), and RNA-dependent RNA polymerase (3D^pol^) (PDB ID: 2E9R). With UCSF Chimera alpha 1.18, water molecules and any co-crystallized ligands were eliminated from the protein structures, polar hydrogen atoms were added, protonation states were designated to the histidine residues, along with assigning partial charges using (AM1-BCC force field) for proteins. The protein structures had been optimized at 500 steepest decent minimization steps on size 0.01 Å and 50 conjugate gradient steps on size 0.01 Å [11].

**Table 1:** Identification of glycyrrhizic acid and chitosan on PubChem database

**Ligand**	**Glycyrrhizic Acid**	**Chitosan**
**SMILES**	C[C@]12CC[C@](C[C@H]1C3=CC(=O)[C@@H]4[C@]5(CC[C@@H](C([C@@H]5CC[C@]4([C@@]3(CC2)C)C)(C)C)O[C@@H]6[C@@H]([C@H]([C@@H]([C@H](O6)C(=O)O)O)O)O[C@H]7[C@@H]([C@H]([C@@H]([C@H](O7)C(=O)O)O)O)O)C)(C)C(=O)O	C([C@@H]1[C@H]([C@@H]([C@H]([C@@H](O1)OC2[C@H](O[C@H](C([C@H]2O)N)OC3[C@H](OC(C(C3O)N)O)CO)CO)N)O)O)O
**PubChem ID**	14982	129662530
**Formula**	C_42_H_62_O_16_	C_18_H_35_N_3_O_13_

#### Active site prediction of proteins

The binding sites of target protein, including amino acid residues, with ligands are the active sites distributed on the protein surface that are responsible for protein functions. These sites acted as the catalytic residues and were visualized through BIOVIA Discovery Studio 2020. Also, 2D interaction structure for each docking process was taken using this software program [12].

**Fig. 2:**
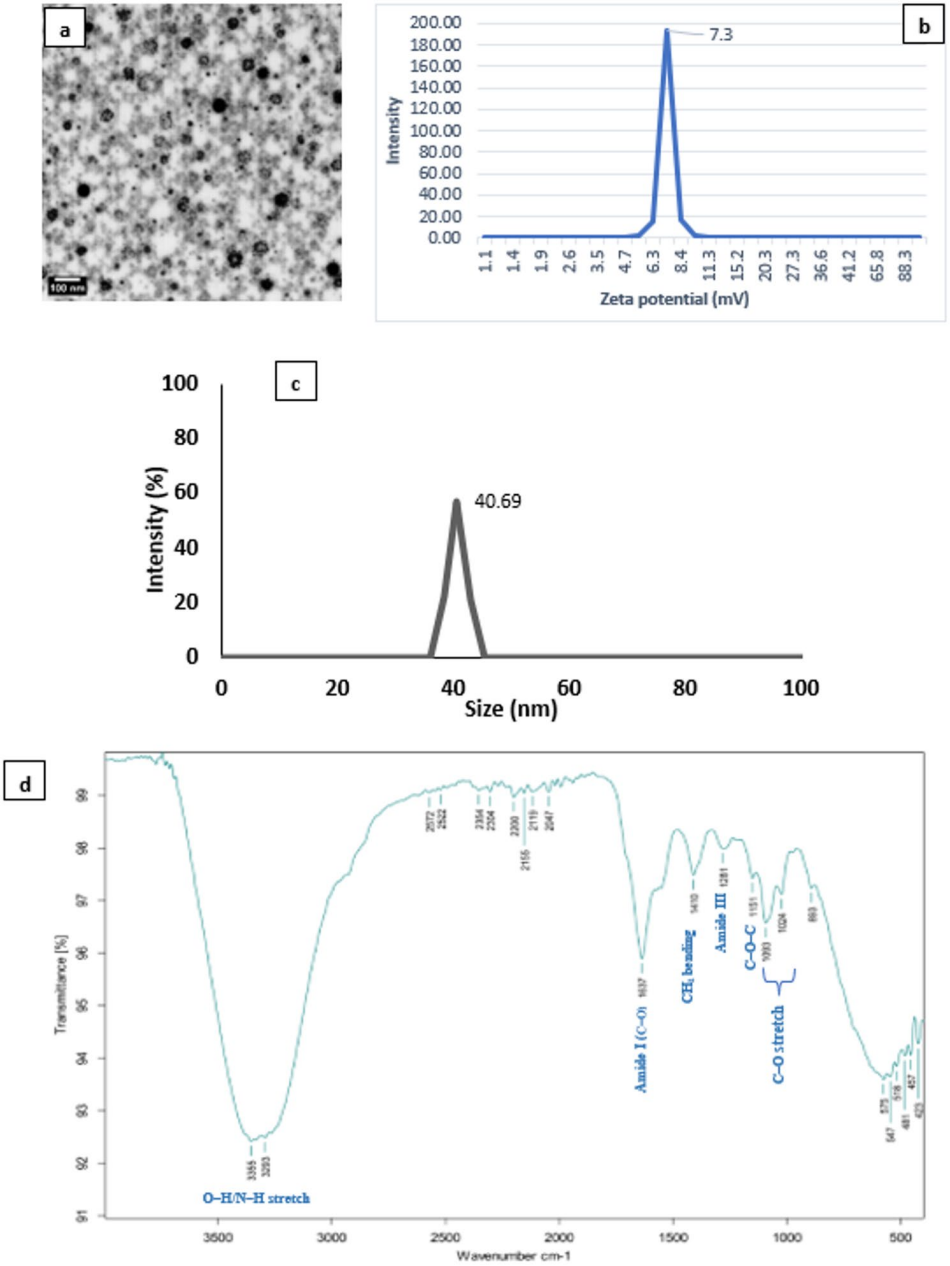
Characterization of Ch-NPs. **a:** TEM of Ch-NPs **b:** Zeta potential of Ch-NPs **c:** DLS of Ch-NPs **d:** FTIR spectrum of Ch-NPs

#### Molecular docking of ligands with target viral proteins

Molecular docking of ligands with proteins was performed using UCSF Chimera alpha 1.18 software. This process demands the target proteins and ligands to be in PDBQT format. Furthermore, binding poses were predicted, binding affinities were calculated to identify key interactions among glycyrrhizic acid and chitosan, and the target proteins, and the ligand binding affinity for each protein was evaluated.

**Table 2:** Binding affinities of GA-NPs with the amino acid residues involved in interactions

**Target FMDV protein**	**Target ** **ligand**	**Binding affinity** **(kcal/mol)**	**Type of interactions**
**3C protease** **(3C** ^**pro**^ **)** **(PDB ID: 2WV4)**	**Glycyrrhizic Acid** **(PubChem CID: 14982)**	-6.732	-van der Waals interaction with ARG A:159, ALA A:163, VAL A:19, CYS A:31, GLU A:20, THR A:27, PHE A:48, LEU A:23, LEU A:47, LEU A:21, TYR A:162 -Carbon-hydrogen interaction with VAL A:28 -Hydrogen bond HIS A:46 -Alkyl and Pi-Alkyl interaction with TYR A:52, LYS A:51, ALA A:29 -Unfavorable interaction with ALA A:160, GLY A:161, ILE A:30, ALA A:49, GLU A:50
**RNA-dependent RNA polymerase** **(3D** ^**pol**^ **)** **(PDB ID: 2E9R)**		-8.974	-van der Waals interaction with PHE X:244, SER X:298, THR X:303, VAL X:215, ALA X:302, ILE X:305, CYS X:300, CYS X:200, LEU X:108, ILE X:189, THR X:115, SER X:304, TYR X:336, GLY X: 337, ASP X:339, ASN X:307, ARG X:179, ASP X:338 -Carbon-hydrogen interactions with SER X:301, MET X:111 -Hydrogen bonds ASP X:245, HIS X:204, ARG X:193, ASP X:109, GLY X:299, ARG X:128, GLU X:112, ALA X:110
**VP1 of FMDV capsid**	**Glycyrrhizic Acid** **(PubChem CID: 14982)**	-8.450	-van der Waals interaction with VAL 1:50, ASP 1:52, PRO 1:190, MET 1:54, VAL 1:129, ARG 1:67, ASN 1:131, GLY 1:132, PRO 1:160, GLN 1:55 -Carbon-hydrogen interactions with THR 1:128 -Hydrogen bonds THR 1:161, ASN 1:164, ARG 1:189, ARG 1:157, TYR 1:130 -Alkyl interaction with LEU 1:159, PRO 1:158 -Unfavorable interaction with ARG 1:189
**VP2 of FMDV capsid**		-8.765	-van der Waals interaction with ILE 2:197, GLN 2:196, ALA 2:194, PRO 2:195, GLY 2:113, THR 2:26, GLN 2:27, THR 2:155, MET 2:154 -Carbon-hydrogen interactions with THR 2:110 -Hydrogen bonds SER 2:28, THR 2:25, SER 2:24, ASN 2:202, GLU 2:108 -Alkyl and Pi-Alkyl interaction with LYS 2:198, TYR 2:200, VAL 2:112
**VP3 of FMDV capsid**		-8.424	-van der Waals interaction with THR 3:178, THR 3:177, GLU 3:176, VAL 3:73, LYS 3:84, ALA 3:83, PRO 3:132, GLU 3:131, ASN 3:179, MET 3:130 -Carbon-hydrogen interactions with ALA 3:75, PHE 3:77 -Hydrogen bonds GLY 3:129, TRP 3:183, ASP 3:78, HIS 3:85, GLN 3:76
**VP4 of FMDV capsid**		-6.438	-van der Waals interaction with GLY 4:16, GLN 4:31, MET 4:27, ASN 4:23, ILE 4:22, SER 4:20, GLY 4:19, ILE 4:21 -Carbon-hydrogen interactions with ASN 4:32 -Hydrogen bonds THR 4:18, GLN 4:28, ASN 4:32, SER 4:15, TYR 4:26, ASN 4:24

## FMD virus strains

Local FMDV strains (O PanAsia-2, A Iran 05, A Africa 2020, A Africa G IV Egypt 2022, A Venezuela, and SAT2/EGY/2012) isolated from calves and were typed and subtyped at the FMD Research Department (FMDRD), and Veterinary Serum and Vaccine Research Institute (VSVRI), Abasia, Cairo. The isolates verified by the World Reference Laboratories, Pirbright, United Kingdom. These viral strains were used in monitoring the virucidal activity of GA-NPs and Ch-NPs. These viruses had the titers of 8.5, 8, 8, 7.5, 7.5, and 7 TCID_50_/ml sequentially and used in monitoring the virucidal activity of GA-NPs and Ch-NPs.

Guinea pig adapted FMD viruses were supplied by the same department and used in the experimental testing of the in vivo antiviral effect of the used nanoparticles.

**Fig. 3:**
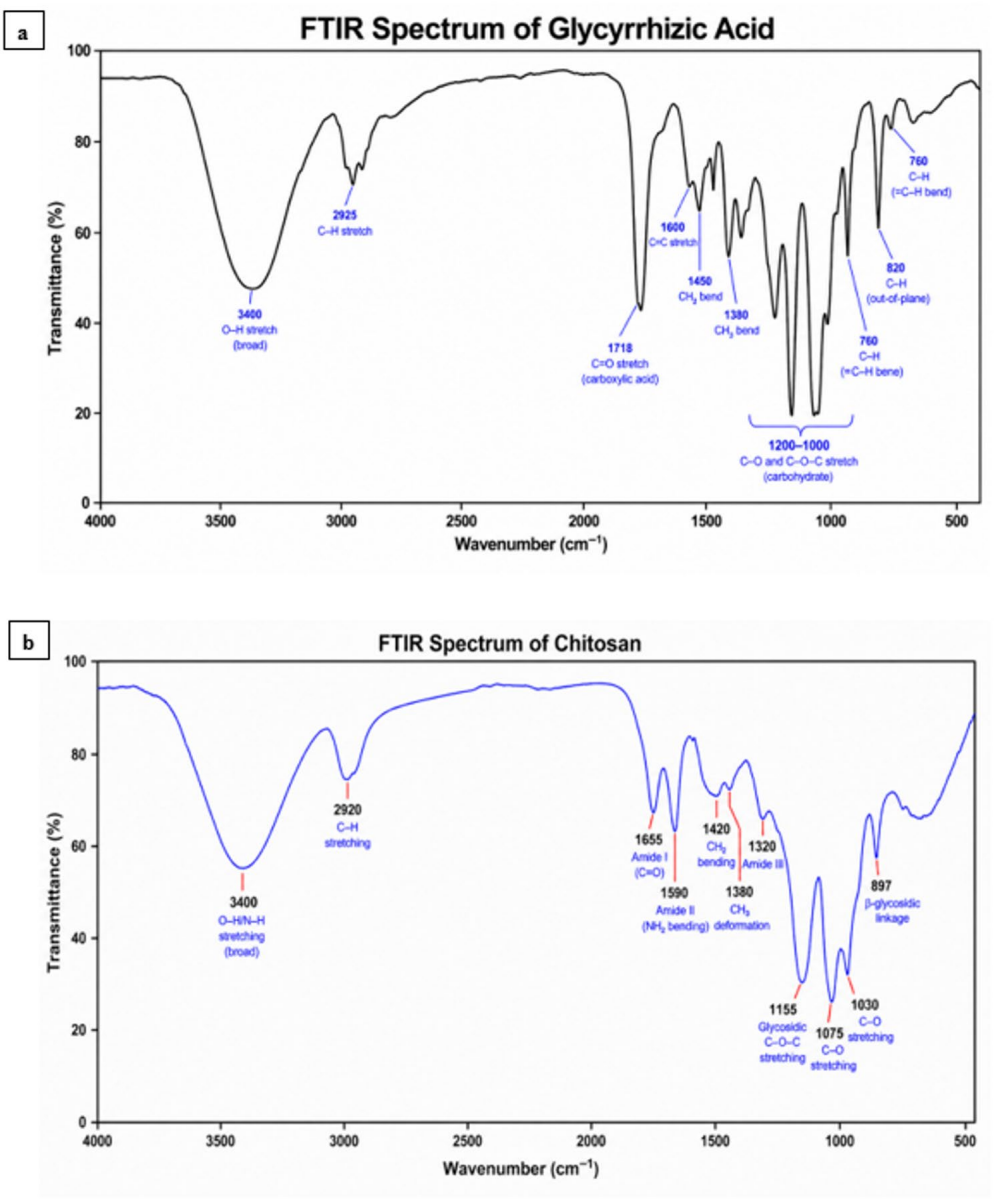
FTIR spectrum. **a: **Glycyrrhizic acid **b:** Chitosan

## Nanoparticles and their preparation

### Chitosan

(CAS number: 9012-76−4), purchased from Sigma-Adrich, was equipped using ionotropic chitosan gelation with sodium tripolyphosphate (TPP) anions. At room temperature, chitosan (0.2%) was combined in ascorbic acid (1%) solution and stirred for 1 h (1000 rpm). A TPP stock solution was prepared by solubilizing 0.03 g of TPP in 11 ml of distilled water. Chitosan nanoparticles were spontaneously assembled when 1 ml of TPP stock solution was added gradually drop by drop to chitosan solution under magnetic stirring (1000 rpm, 1 h) at ambient temperature. The chitosan nanoparticles were then sonicated for an hour to assure their nano size [13].

**Table 3:** Binding affinities of Ch-NPs with the amino acid residues involved in interactions

**Target FMDV protein**	**Target ** **ligand**	**Binding affinity ** **(kcal/mol)**	**Type of interactions**
**3C protease** **(3C** ^**pro**^ **)** **(PDB ID: 2WV4)**	**Chitosan** **(PubChem CID: 129662530)**	-5.796	-van der Waals interaction with GLY A:161, ALA A:160, LEU A:47, CYS A:31, ASP A:144, SER A:182, ALA A:183, ALA A:163, THR A:27 -Carbon-hydrogen interactions with ALA A:29, VAL A:28 -Hydrogen bonds ILE A:30, ASP A:123, GLU A:50, HIS A:46
**RNA-dependent RNA polymerase** **(3D** ^**pol**^ **)** **(PDB ID: 2E9R)**		-7.132	-van der Waals interaction with VAL X:181, PHE X:244, LYS X:164, SER X:242, LYS X:177, SER X:58, THR X:178, LYS X:59, ILE X:56, ARG X:168, PHE X:57, TYR X:336, ASN X:307, GLY X:337, THR X:303, SER X:298 -Carbon-hydrogen interactions with VAL X:55, ASP X:338, ASP X:245 -Hydrogen bonds SER X:304, ARG X:179, ILE X:180, VAL X:55, ALA X:243
**VP1 of FMDV capsid**		-5.769	-van der Waals interaction with ARG 1:189, LEU 1:192, LEU 1:191, THR 1:68, ALA 1:64, VAL 1:129, GLN 1:55, PRO 1:158, LEU 1:169 -Carbon-hydrogen interactions with THR 1:128, ARG 1:67 -Hydrogen bonds SER 1:58, PRO 1:190, ARG 1:67, ARG 1:157, MET 1:54, THR 1:128
**VP2 of FMDV capsid**	**Chitosan** **(PubChem CID: 129662530)**	-5.788	-van der Waals interaction with THR 2:23, SER 2:24, VAL 2:112, MET 2:154, THR 2:26, THR 2:155, VAL 2:109, TYR 2:200 -Carbon-hydrogen interactions with THR 2:25, LYS 2:63 -Hydrogen bonds ASN 2:202, ALA 2:201, THR 2:110, SER 2:28, GLN 2:27 -Salt bridge GLU 2:108
**VP3 of FMDV capsid**		-7.173	-van der Waals interaction with VAL 3:180, ASN 3:179, ALA 3:175, HIS 3:85, PHE 3:77, GLN 3:76, ASP 3:173, ALA 3:82, LEU 3:79, GLN 3:181, THR 3:170, ALA 3:174 -Carbon-hydrogen interactions with ALA 3:83, ASP 3:78 -Hydrogen bonds ALA 3:171, TYR 3:169, SER 3:172, LYS 3:84, GLU 3:176, THR 3:177, THR 3:178, SER 3:80 -Salt bridge ASP 3:78
**VP4 of FMDV capsid**		-4.887	-van der Waals interaction with GLY 4:19, MET 4:27, TYR 4:26, ASN 4:32, GLN 4:31 -Carbon-hydrogen interactions with ASN 4:23, ASN 4:24 -Hydrogen bonds THR 4:18, SER 4:20, ILE 4:22, GLN 4:28 -Unfavorable positive-positive interaction with SER 4:15

### Glycyrrhizic acid

Was obtained from Sigma-Aldrich company; 1 mg of glycyrrhizic acid was dissolved in 1 ml absolute ethanol and sonicated (XUBA3Analogue Ultrasonic Bath, Grant Company), at 25 °C, with an ultrasonic power and frequency of 50 kHz for an hour. The concentration was prepared and enforced to be 2 mg/ml nanoform [14].

**Fig. 4:**
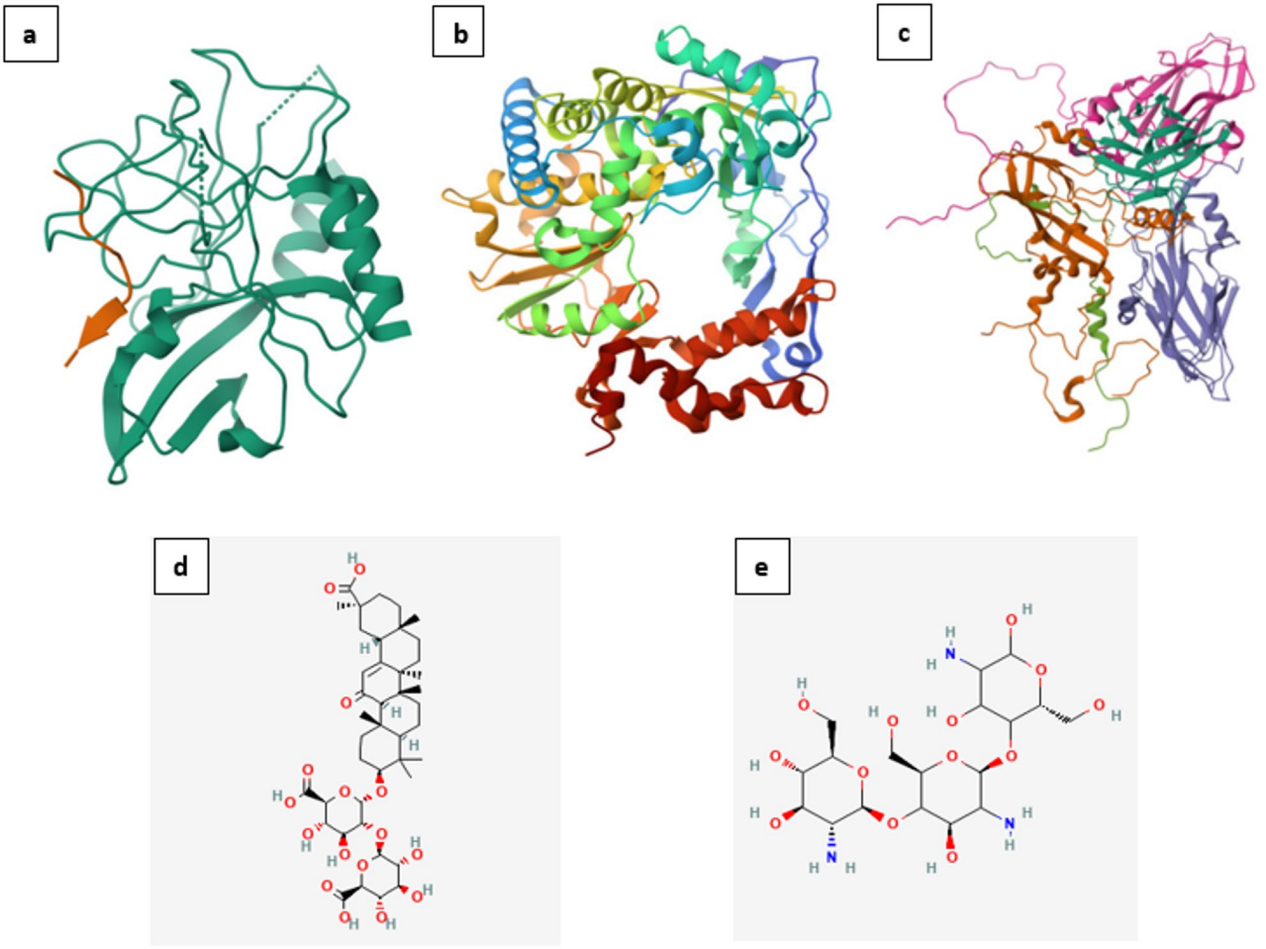
3D structure of FMD viral protein and 2D structure of ligands. **a:** 3C protease (3C^pro^) **b:** RNA-dependent RNA polymerase (3D^pol^) **c:** VP1, VP2, VP3, & VP4 **d:** glycyrrhizic acid **e:** chitosan

## Characterization of nanoparticles

### Dynamic light scattering (DLS)

Measurement of size distribution and zeta potential of GA-NPs and Ch-NPs were executed at Faculty of Science, Cairo University, using a dynamic light scattering (DLS) approach using Zetasizer Nano ZS (Malvern Instruments, UK) at room temperature. Prior to measurement, 30 µl of the nanoparticles were water-diluted with 3 ml at 25 °C. The mean of the Z average of three independent batches indicates the particle size of the nanoparticles [14].

**Table 4:** In vitro cytotoxicity assay of GA-NPs and Ch-NPs on BHK cells

**Tested dilution**	**Nanoparticles’ concentration **	**Cytotoxicity % **
**Un-diluted**	2 mg/ml	0
**1/10**	2 x 10^-1^mg/ml	0
**1/100**	2 x 10^-2^ mg/ml	0

### Transmission electron microscopy (TEM) Analysis

For TEM examination, a drop of the nanoparticles’ solution was settled as a monolayer on an amorphous carbon-supported copper grids and left to dry up by permitting water evaporation under ambient conditions. At Desert Research Center, Cairo, electron micrographs were taken using a JEOL GEM-1010 transmission electron microscope at 70 kV [13].

### Fourier transform infrared spectroscopy (FT-IR)

According to [15], the chemical structures of GA-NPs and Ch-NPs were interpreted with FT-IR (Bruker Vertex 70, Germany) with the IR fingerprints registered among 4000–400 cm^− 1^ using transmittance modes. This analysis implemented at Central Laboratory, Faculty of Science, Ain Shams University.

**Fig. 5:**
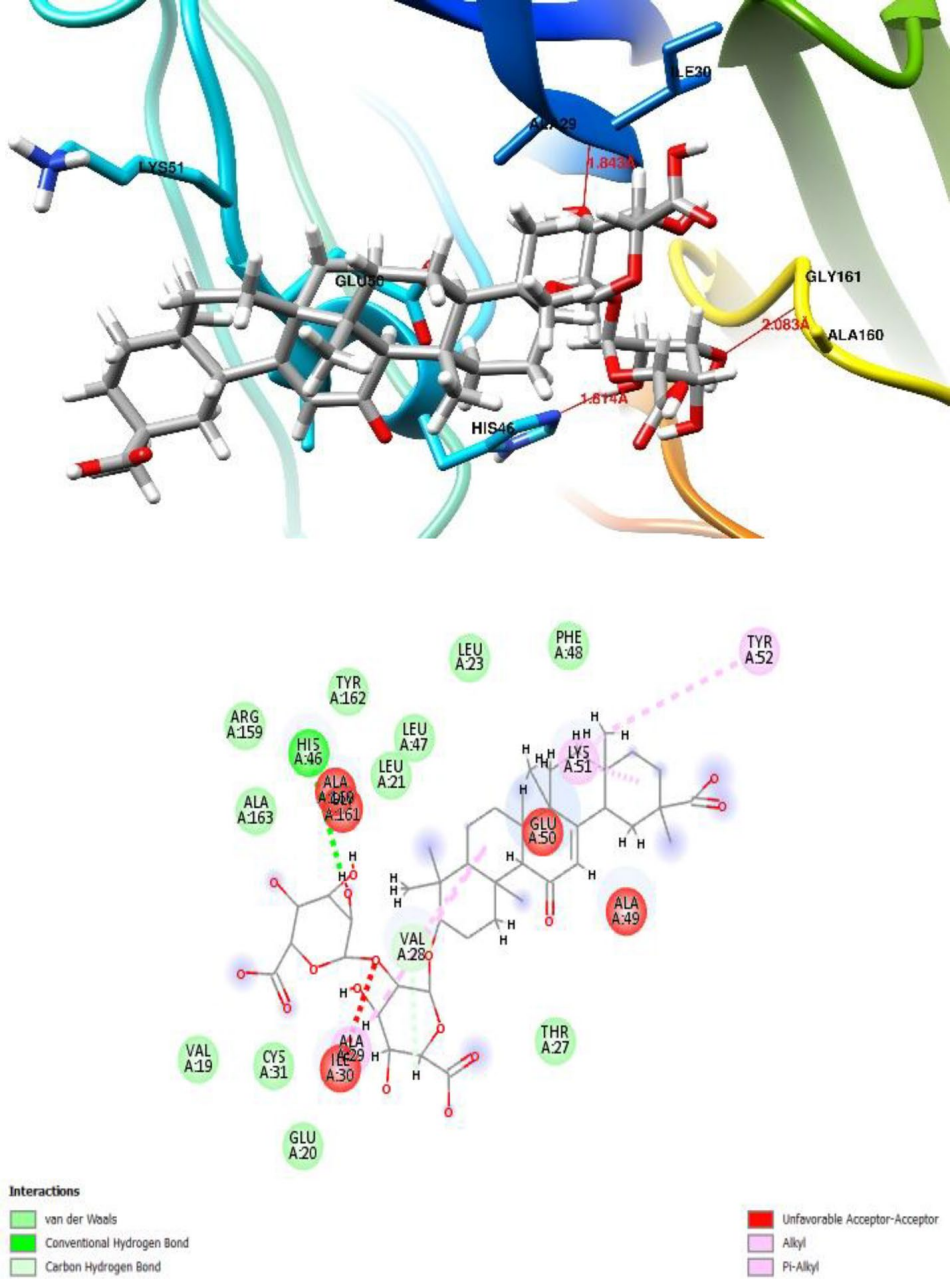
3D and 2D interaction of glycyrrhizic acid within the active sites of 3C^pro^

## Cell culture

Baby Hamster Kidney cell line (BHK_21_ clone_13_) was kindly provided by the Animal Research Institute, Pirbright, UK and propagated at FMDRD. These cells were maintained and propagated as described by [16]; using MEM supplied by Gibco (G80 Gibco Limited, Parsley, U.K.) supplemented with 10% new born calf serum and antibiotics (100 µgs of streptomycin and 100 IU of penicillin-G sodium/ml). These cells were used for study the safety of GA-NPs and Ch-NPs, their antiviral activity, and virus propagation and titration.

## In vitro cytotoxicity test

In vitro biosafety studies of chitosan and glycyrrhizic acid nanoparticles were performed using BHK cells seeded into 96-well culture plates at a density of 5 × 10^3^ cells/well. After 12 h, cells were exposed to treatment with the tested material at different concentrations of 2, 2 × 10^− 1^, and 2 × 10^− 2^ mg/ml, and incubated at 37 °C for 24 h and 5% CO_2_. Each experiment was performed in triplicate. Cytotoxicity was determined by microscopical examination and viable cells were calculated using cell counter.

**Fig. 6:**
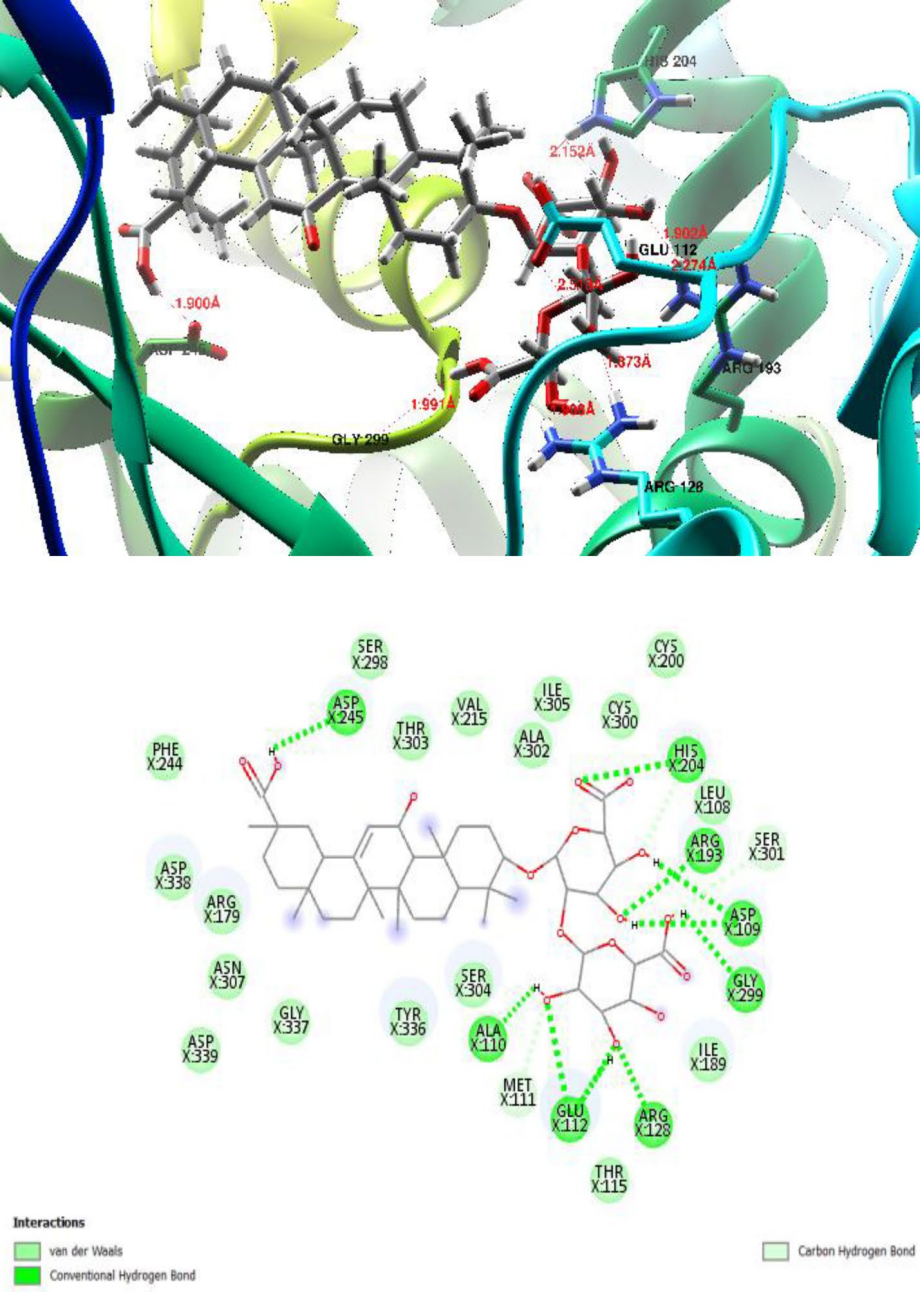
3D and 2D interaction of glycyrrhizic acid within the active sites of 3D^pol^

## In vivo toxicity test

### In Guinea pigs

Twenty-four guinea pigs were grouped into two sets (12 G. pigs/set for Ch-NPs, and 12 G. pigs/set for GA-NPs) to evaluate the in vivo toxicity of GA-NPs and Ch-NPs. Each set was sectioned into 4 groups (3 G. pigs/group). Three groups received intraperitoneal (I/P) injections of 0.4 ml/animal of serial ten-fold dilutions of either Ch-NPs or GA-NPs, while the fourth group received sterile normal saline as test control. All animals were received balanced ration and sufficient water and kept under hygienic measures and daily clinical surveillance for detection of any abnormal post inoculation reactions. The safe concentration was considered as one which didn’t cause any post inoculation reaction as stated by [17].

**Table 5:** In vivo toxicity% of GA-NPs and Ch-NPs in mice

**Inoculated dilution**	**Nanoparticles’ concentration** **(mg/ml)**	**Number of experimental mice**			**Toxic reaction** **%**
		**Total**	**Reacted**	**Non-reacted**	
**Un-diluted**	2	3	0	3	0
**1/10**	2 x 10^-1^	3	0	3	0
**1/100**	2 x 10^-2^	3	0	3	0
**Control**	Saline	3	0	3	0

### In Baby Mice

According to [17, 18], a total of twenty-four baby Swiss mice (2–3-days old) were divided into 2 groups (12 mice for Ch-NPs, and 12 mice for GA-NPs). Each group was divided into 4 subgroups (3 animals/group) and was inoculated intraperitoneally (I/P) with 0.2 ml/mouse of diverse dilutions of Ch-NPs and GA-NPs (three mice for each dilution). Besides that, a control group of baby mice inoculated with saline. After 48 h post inoculation, all the baby mice examined to assess the effect of nanoparticles. Mice death refers that this concentration of nanoparticles is toxic.

**Table 6:** In vivo toxicity% of GA-NPs and Ch-NPs in G. pigs

**Inoculated dilution**	**Nanoparticles’ concentration** **(mg/ml)**	**Number of experimental G. pigs**			**Toxic reaction** **%**
		**Total**	**Reacted**	**Non-reacted**	
**Undiluted**	2	3	0	3	0
**1/10**	2 x 10^-1^	3	0	3	0
**1/100**	2 x 10^-2^	3	0	3	0
**Control**	Saline	3	0	3	0

## Antiviral assay

### Determination of virus titer in tissue culture

It was done as described by [19] where serial ten folds dilutions of FMDV were prepared in tissue culture plates using maintenance medium, 25 µl/well, from each dilution a set of 4 wells were inoculated on BHK cells, control non-infected cells were inoculated with 25 µl of maintenance medium then the plate was incubated at 37 °C for 2 days and observed for the cytopathic effect (CPE) and compared with the control non-infected cells. Finally, the virus titer was expressed as log_10_ TCID_50_ as described by [20]. Virus titration was performed in multiple replicate wells to determine the TCID₅₀ endpoint.

**Fig. 7:**
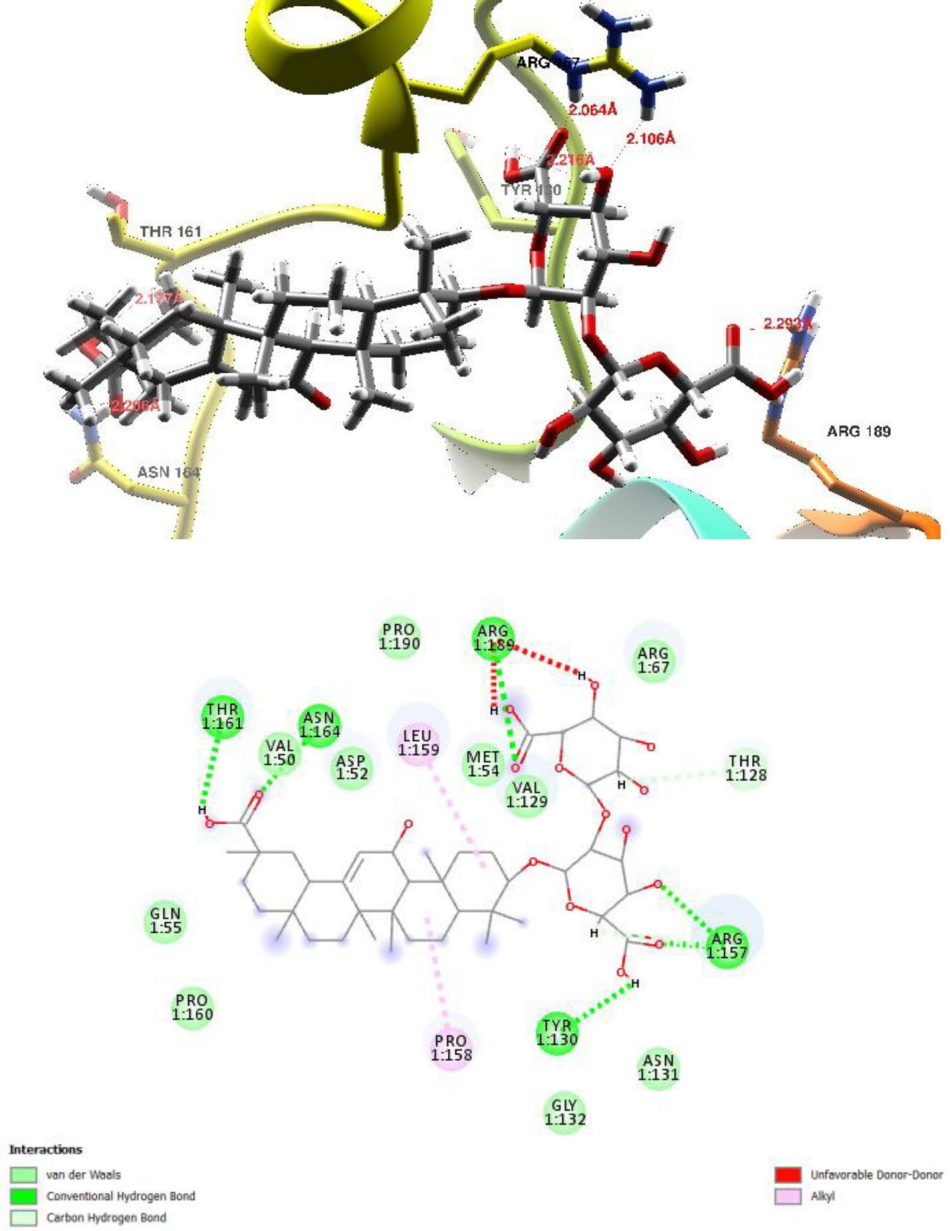
3D and 2D interaction of glycyrrhizic acid within the active sites of VP1 of FMDV capsid

### In vitro antiviral assay

It was done according to [21, 22]. A mixture of 25 µl containing 100 TCID_50_/ml of the used virus with 25 µl of each ten-fold dilution of nanoparticles was kept at 37^o^ C for 30 min then inoculated in each of 6 wells of cultured BHK plates. The test included normal and virus infected cell controls and the plates were examined, under an inverted light microscope, for viral cytopathic effect (CPE). The existence of 100% CPE in virus infected control means negative antiviral effect. The antiviral assay was performed once using six technical replicate wells for each treatment. No independent biological replicate experiments were conducted; therefore, statistical analysis was not applicable.

The percentage of virucidal activity was determined as follows:

$$\text{Virucidal activity} (\%) = \frac{\text{Virus titer}_\mathrm{control}-\text{Virus titer}_\mathrm{treated}}{\text{Virus titer}_\mathrm{control}} \times 100$$

## In vivo antiviral assay

### In baby mice

According to [17, 18], a total of 330 baby Swiss mice (2–3-days old) were divided into 2 groups (165 mice for Ch-NPs, and 165 mice for GA-NPs). Each group was divided into 3 dilution groups. Each dilution group was divided into 6 subgroups (3 animals/group). Within each subgroup, three mice were inoculated intraperitoneally (I/P) with 0.2 ml of a mixture containing one of the tested FMDV types (10^7^ ID_50_/ml) and different dilutions of Ch-NPs and GA-NPs. A virus control group of baby mice was inoculated with the corresponding FMDV only and another group was kept without inoculation as a control. After 48 h post inoculation, all baby mice examined to evaluate the antiviral action of nanoparticles against FMDV serotypes. Paralysis of the hind limbs or death of infected mice means positive FMDV infection while death after 24 h is considered non-specific result.

**Fig. 8:**
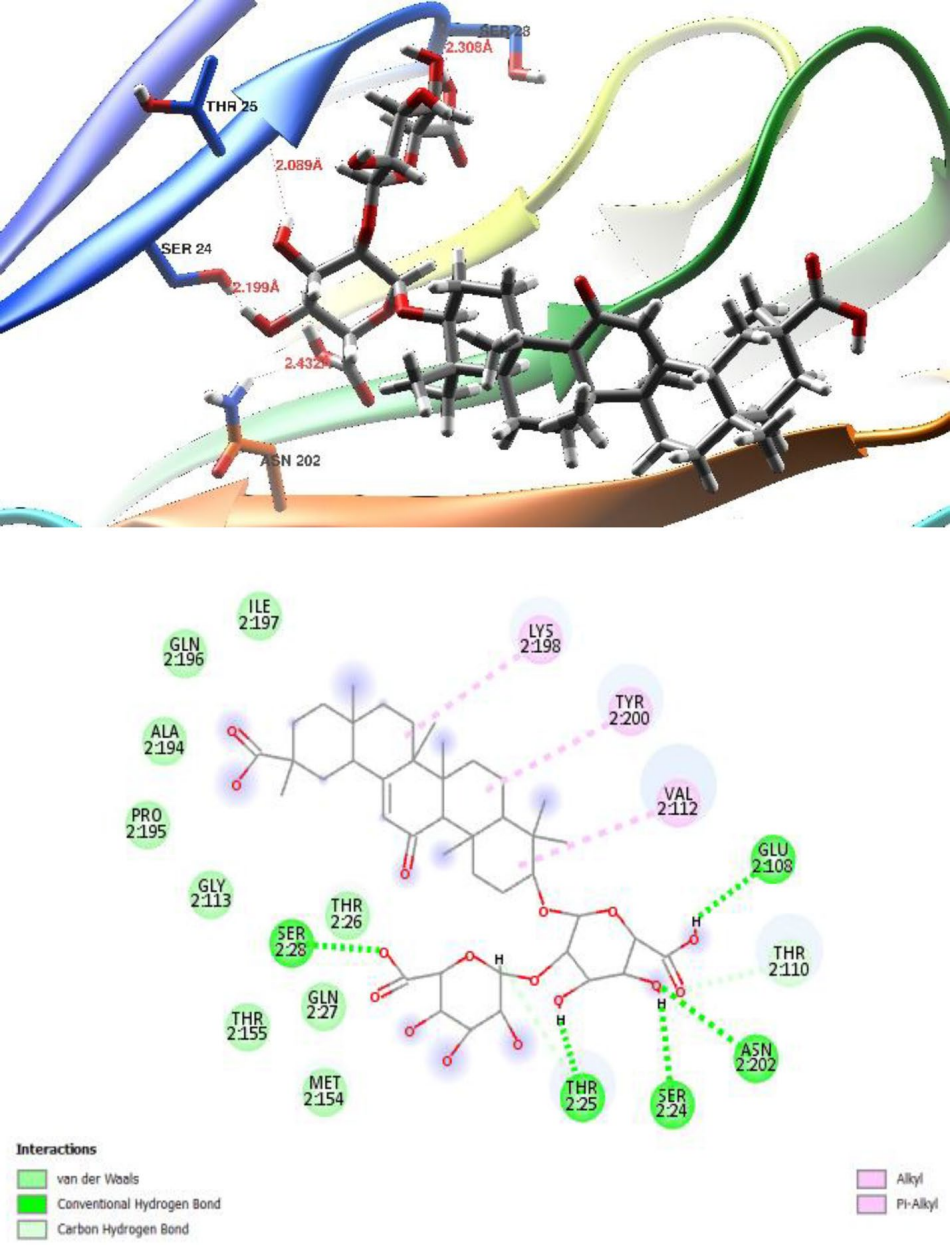
3D and 2D interaction of glycyrrhizic acid within the active sites of VP2 of FMDV capsid

### In G. pigs

As described by [23], 330 G. pigs were divided into 2 groups (165 G. pigs for Ch-NPs, and 165 G. pigs for GA-NPs). Each group was divided into 3 dilution groups. Each dilution group was divided into 6 subgroups (3 animals/group). Within each subgroup, three G. pigs were inoculated in the foot pad with 0.4 ml of a mixture containing one of the tested FMDV types (10^7^ ID_50_/ml) and different dilutions of Ch-NPs and GA-NPs. Non inoculated and virus inoculated groups were included as test controls. All animals were kept under daily clinical observation to detect any signs of FMD virus infection.

**Table 7:** In vitro virucidal assay of GA-NPs and Ch-NPs concentrations against FMDV on BHK cells

**Tested dilution**	**Nanoparticles’ ** **Concentration** ** (mg/ml)**	**FMDV serotype**	**Virus sample** **Number**	**Virus titer (log**_**10**_ ** TCID**_**50**_ **/ml)**				**% of virucidal action**
				**Starting titer**	**Mean starting titer**	**Residual Virus titer**	**Reduction of virus titer**	
**Undiluted**	2	O	V1	7.5	8	0	8	100
			V2	8		0		
			V3	8.5		0		
		A Iran 05	V4	7	7.5	0	7.5	100
			V5	7.5		0		
			V6	8		0		
		A Africa 2020	V7	6.5	7	0	7	100
			V8	7		0		
			V9	7.5		0		
		A Africa G IV Egypt 2022	V10	6.5	7	0	7	100
			V11	7		0		
			V12	7.5		0		
		A Venezuela	V13	7	7.5	0	7.5	100
			V14	7.5		0		
			V15	8		0		
		SAT2	V16	6	6.5	0	6.5	100
			V17	6.5		0		
			V18	7		0		
		control	V19	7.5	7.5	7.5	0	-
**1/10**	2 x 10^-1^	O	V1	7.5	8	0	8	100
			V2	8		0		
			V3	8.5		0		
		A Iran 05	V4	7	7.5	0	7.5	100
			V5	7.5		0		
			V6	8		0		
		A Africa 2020	V7	6.5	7	0	7	100
			V8	7		0		
			V9	7.5		0		
		A Africa G IV Egypt 2022	V10	6.5	7	0	7	100
			V11	7		0		
			V12	7.5		0		
		A Venezuela	V13	7	7.5	0	7.5	100
			V14	7.5		0		
			V15	8		0		
		SAT2	V16	6	6.5	0	6.5	100
			V17	6.5		0		
			V18	7		0		
		control	V19	7.5	7.5	7.5	0	-
**1/100**	2 x 10^-2^	O	V1	7.5	8	1.5	8	81.25
			V2	8		2		
			V3	8.5		2.5		
		A Iran 05	V4	7	7.5	0	7	100
			V5	7.5		0		
			V6	8		0		
		A Africa 2020	V7	6.5	7	0	7	100
			V8	7		0		
			V9	7.5		0		
		A Africa G IV Egypt 2022	V10	6.5	7	0.5	5.5	92.86
			V11	7		1		
			V12	7.5		1.5		
		A Venezuela	V13	7	7.5	1	6	80
			V14	7.5		1.5		
			V15	8		2		
		SAT2	V16	6	6.5	0.5	5	92.31
			V17	6.5		0.5		
			V18	7		1		
		control	V19	7.5	7.5	7.5	0	-

## Results

### Characterization of GA-NPs and Ch-NPs

The surface properties of GA-NPs and Ch-NPs were demonstrated by TEM analysis as in Fig. (1a, 2a). They were spherical particles with sizes extending from around 20–80 nm for GA-NPs and 10–40 nm with some particles reaching up to 70 nm for Ch-NPs, with PDI of 0.182 indicating a narrow and homogeneous particle size distribution. Compatible with TEM, GA-NPs show average particle size of 72.22 nm with negative surface charge of −14.25 mV (Fig. (1: b, c)). Whereas Ch-NPs have positive surface charge of 7.3 mV with average particle size of 40.69 nm as shown in Fig. (2: b, c).

**Fig. 9:**
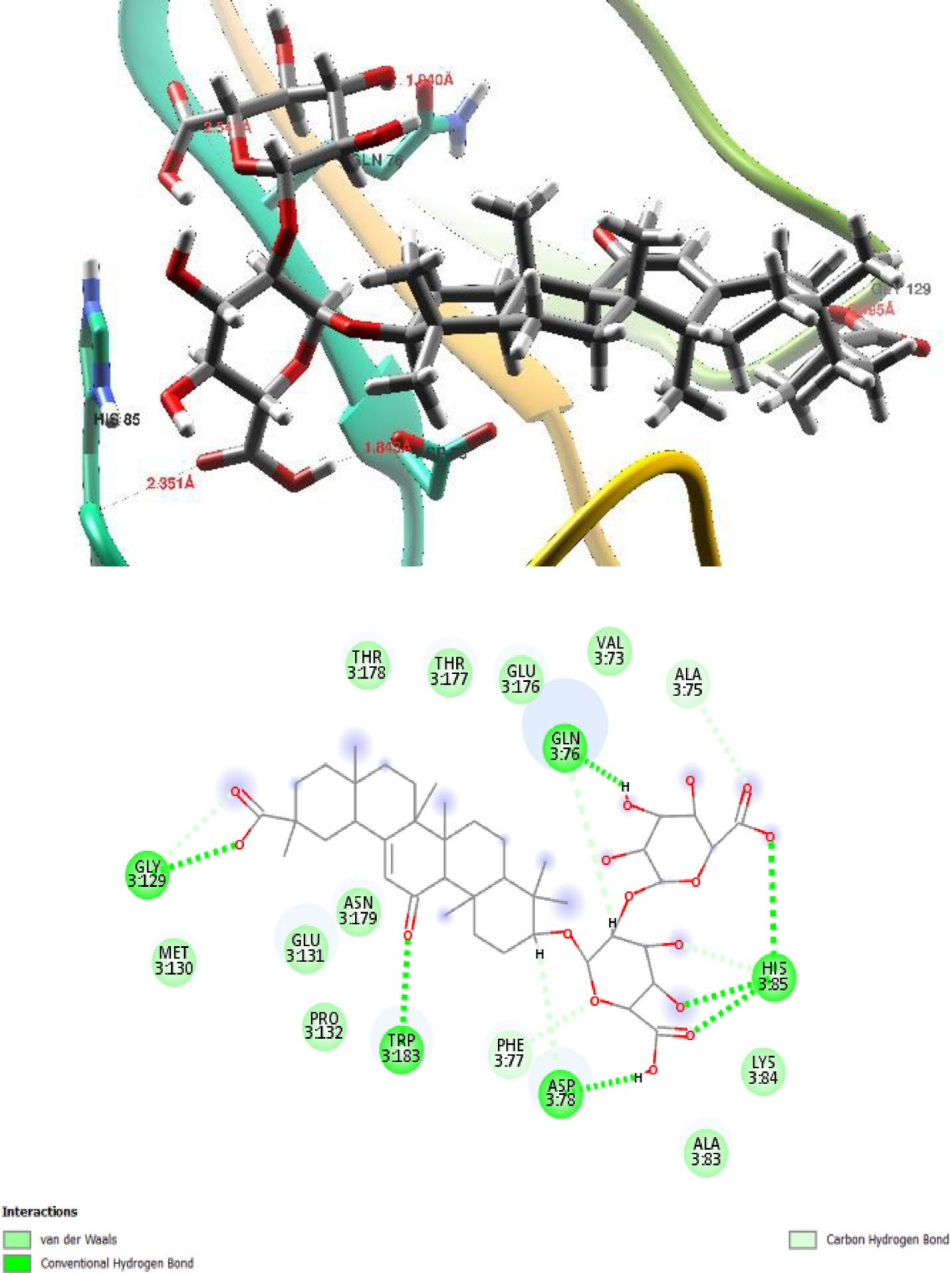
3D and 2D interaction of glycyrrhizic acid within the active sites of VP3 of FMDV capsid

The FTIR spectra of pure glycyrrhizic acid and pure chitosan are presented in Fig. (3) whereas their nanoparticles are illustrated in Fig. (1d, 2 d). GA-NPs showed a broad O–H stretching band that shifted from 3400 to 3332 cm⁻¹. The carbonyl band at 1718 cm⁻¹ shifted toward 1650 cm⁻¹ with reduced intensity. Minor shifts were also observed in the C–H stretching region (2925 cm⁻¹ to 2866 cm⁻¹), while intensity changes occurred in the C–O/C–O–C region (1200–1000 cm⁻¹). Compared with pure chitosan, Ch-NPs FTIR spectrum exhibited shifts in the O–H/N–H (3400 to 3355–3293 cm⁻¹), Amide I (1655 to 1637 cm⁻¹), Amide III (1320 to 1281 cm⁻¹), and C–O/C–O–C bands, together with reduced Amide II intensity. No new absorption bands were detected in both nanoparticles’ spectrum.

**Table 8:** Virucidal assay of GA-NPs and Ch-NPs concentration against FMDV in mice

**Tested** **dilution**	**Nanoparticles’ ** **Concentration** ** (mg/ml)**	**FMDV serotype**	**Virus sample number**	**Number of experimental mice**			**Virucidal action** **%**
				** Total number **	**Number of infected **	**Number of survived**	
**undiluted**	2	O	V1	3	0	3	100
			V2	3	0	3	
			V3	3	0	3	
		A Iran 05	V4	3	0	3	100
			V5	3	0	3	
			V6	3	0	3	
		A Africa 2020	V7	3	0	3	100
			V8	3	0	3	
			V9	3	0	3	
		A Africa G IV Egypt 2022	V10	3	0	3	100
			V11	3	0	3	
			V12	3	0	3	
		A Venezuela	V13	3	0	3	100
			V14	3	0	3	
			V15	3	0	3	
		SAT2	V16	3	0	3	100
			V17	3	0	3	
			V18	3	0	3	
		Control	V19	3	3	0	-
**1/10**	2 x 10^-1^	O	V1	3	0	3	100
			V2	3	0	3	
			V3	3	0	3	
		A Iran 05	V4	3	0	3	100
			V5	3	0	3	
			V6	3	0	3	
		A Africa 2020	V7	3	0	3	100
			V8	3	0	3	
			V9	3	0	3	
		A Africa G IV Egypt 2022	V10	3	0	3	100
			V11	3	0	3	
			V12	3	0	3	
		A Venezuela	V13	3	0	3	100
			V14	3	0	3	
			V15	3	0	3	
		SAT2	V16	3	0	3	100
			V17	3	0	3	
			V18	3	0	3	
		Control	V19	3	3	0	-
**1/100**	2 x 10^-2^	O	V1	3	1	2	66.7
			V2	3	1	2	
			V3	3	1	2	
		A Iran 05	V4	3	2	1	44.4
			V5	3	2	1	
			V6	3	2	1	
		A Africa 2020	V7	3	1	2	66.7
			V8	3	1	2	
			V9	3	1	2	
		A Africa G IV Egypt 2022	V10	3	0	3	100
			V11	3	0	3	
			V12	3	0	3	
		A Venezuela	V13	3	1	2	77.8
			V14	3	1	2	
			V15	3	1	2	
		SAT2	V16	3	0	3	100
			V17	3	0	3	
			V18	3	0	3	
		Control	V19	3	3	0	-

## In silico study

### Molecular docking

**Fig. 10:**
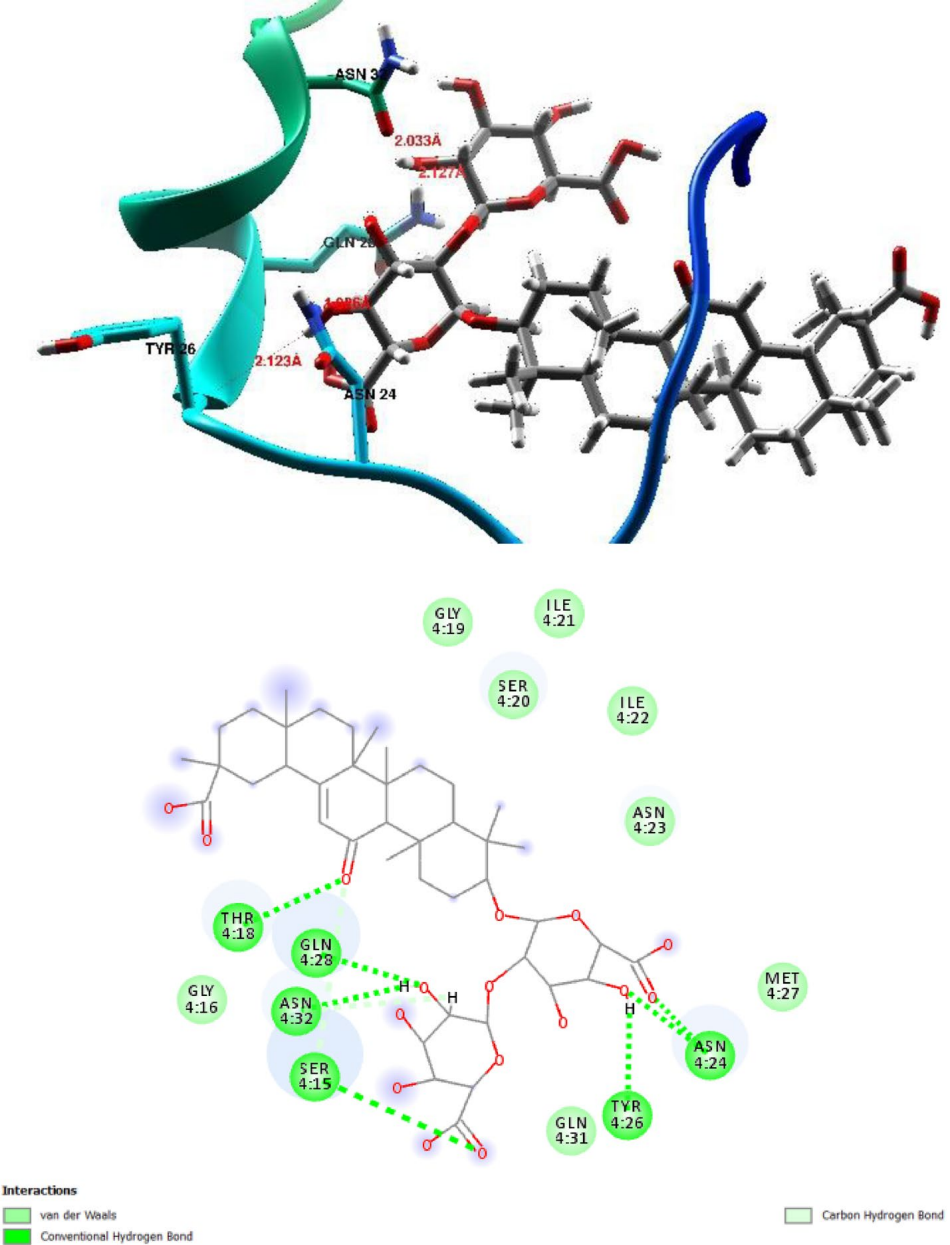
3D and 2D interaction of glycyrrhizic acid within the active sites of VP4 of FMDV capsid

**Fig. 11:**
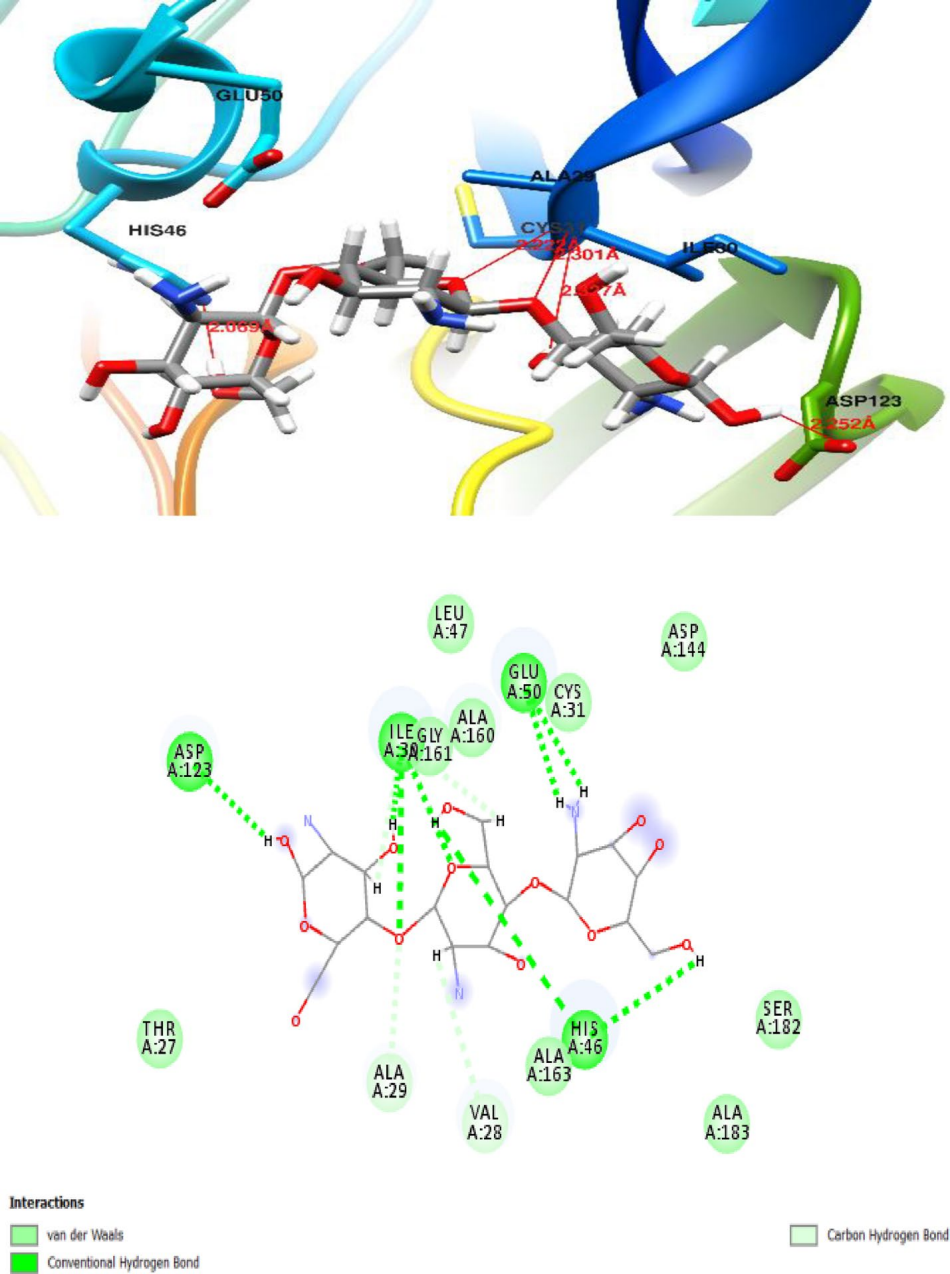
3D and 2D interaction of chitosan within the active sites of 3C^pro^

**Fig. 12:**
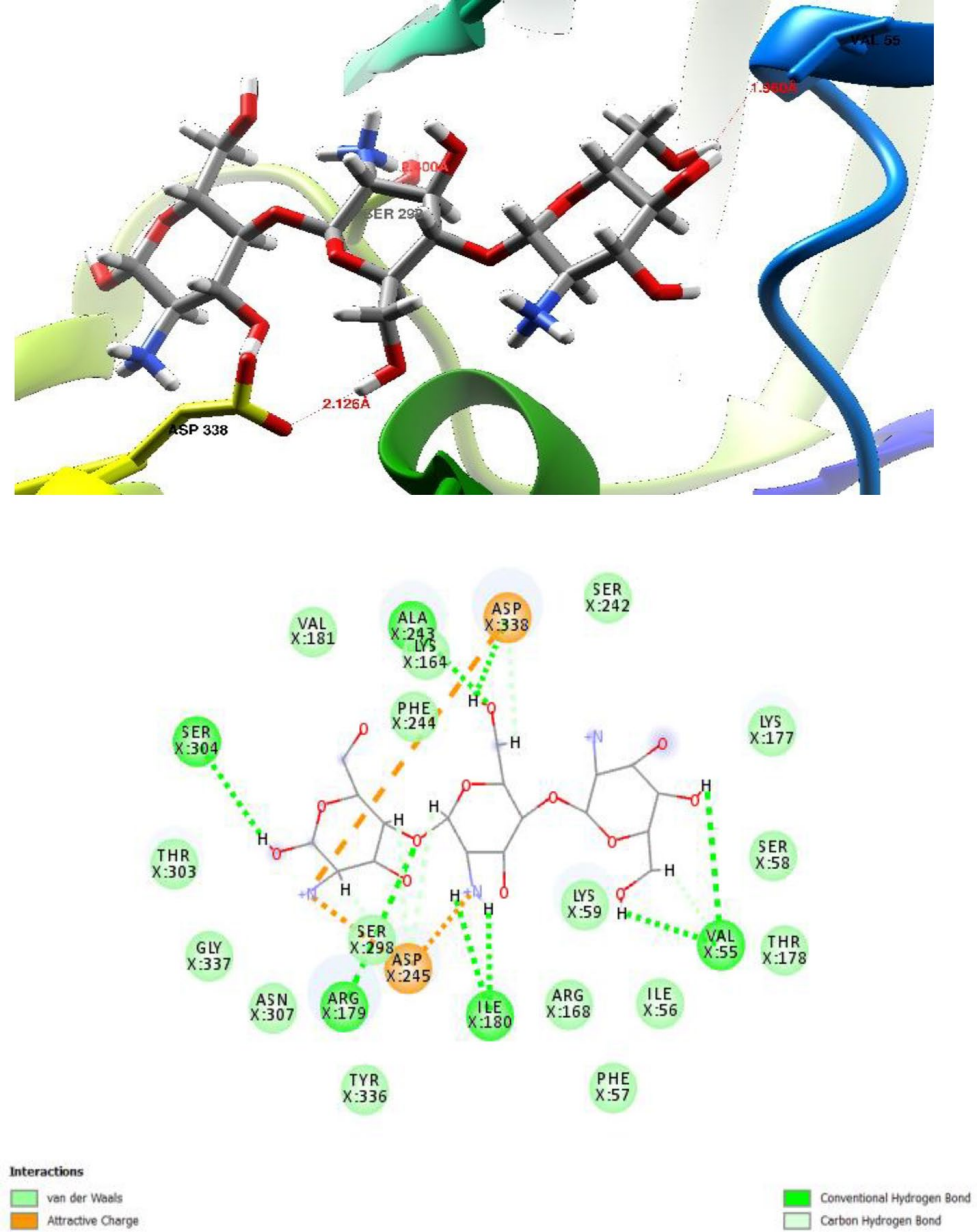
3D and 2D interaction of chitosan within the active sites of 3D^pol^

**Fig. 13:**
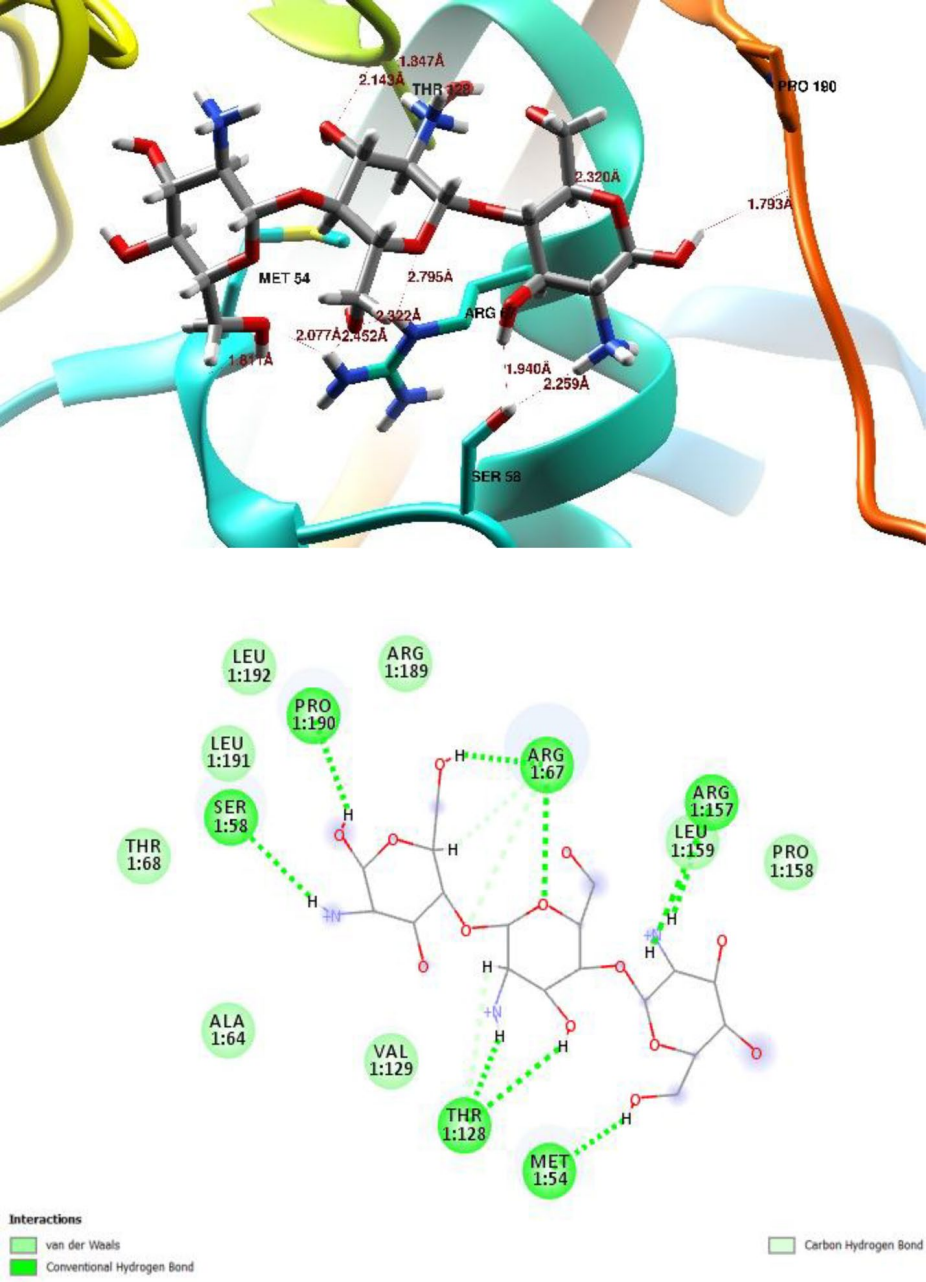
3D and 2D interaction of chitosan within the active sites of VP1 of FMDV capsid

**Fig. 14:**
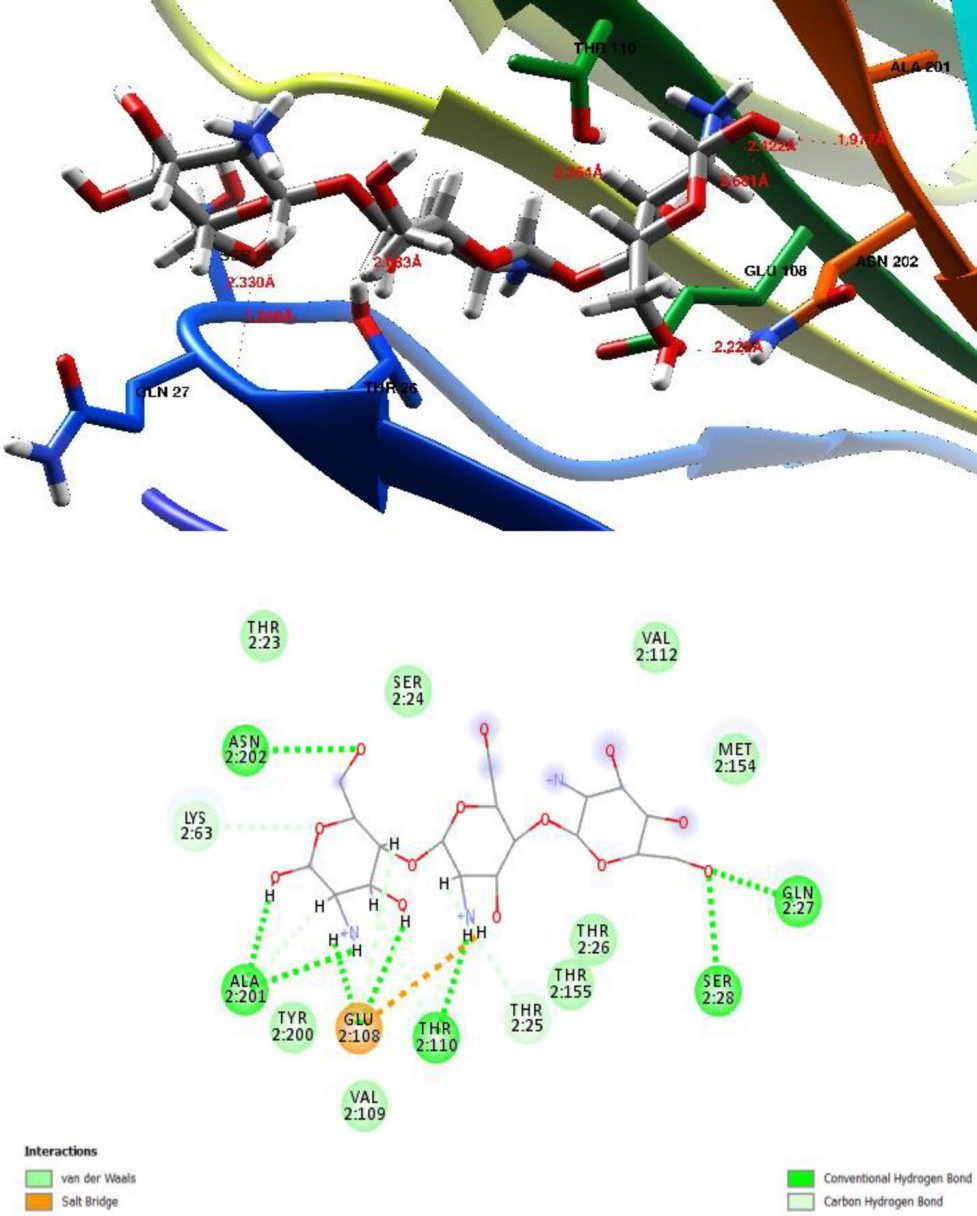
3D and 2D interaction of chitosan within the active sites of VP2 of FMDV capsid

**Fig. 15:**
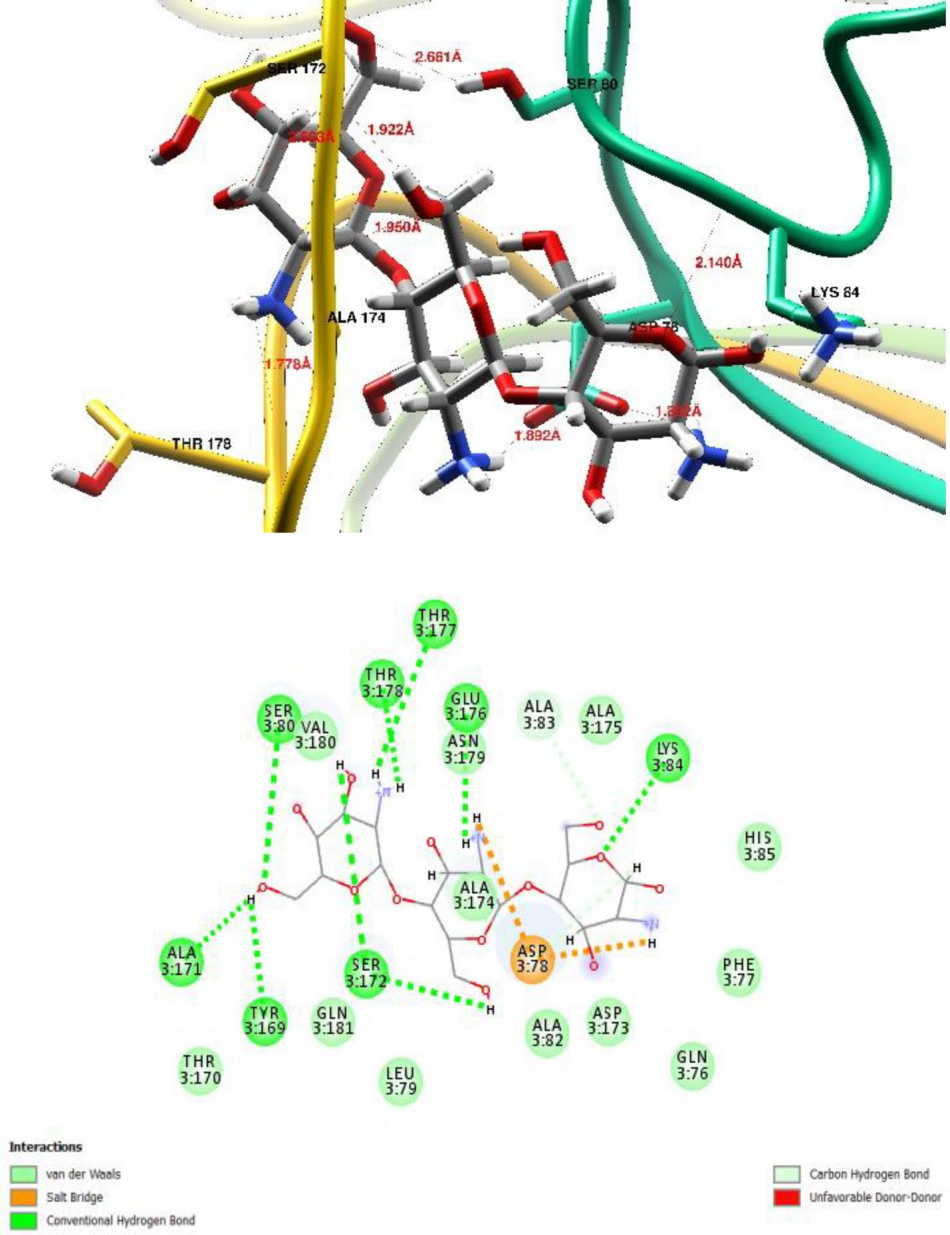
3D and 2D interaction of chitosan within the active sites of VP3 of FMDV capsid

**Fig. 16:**
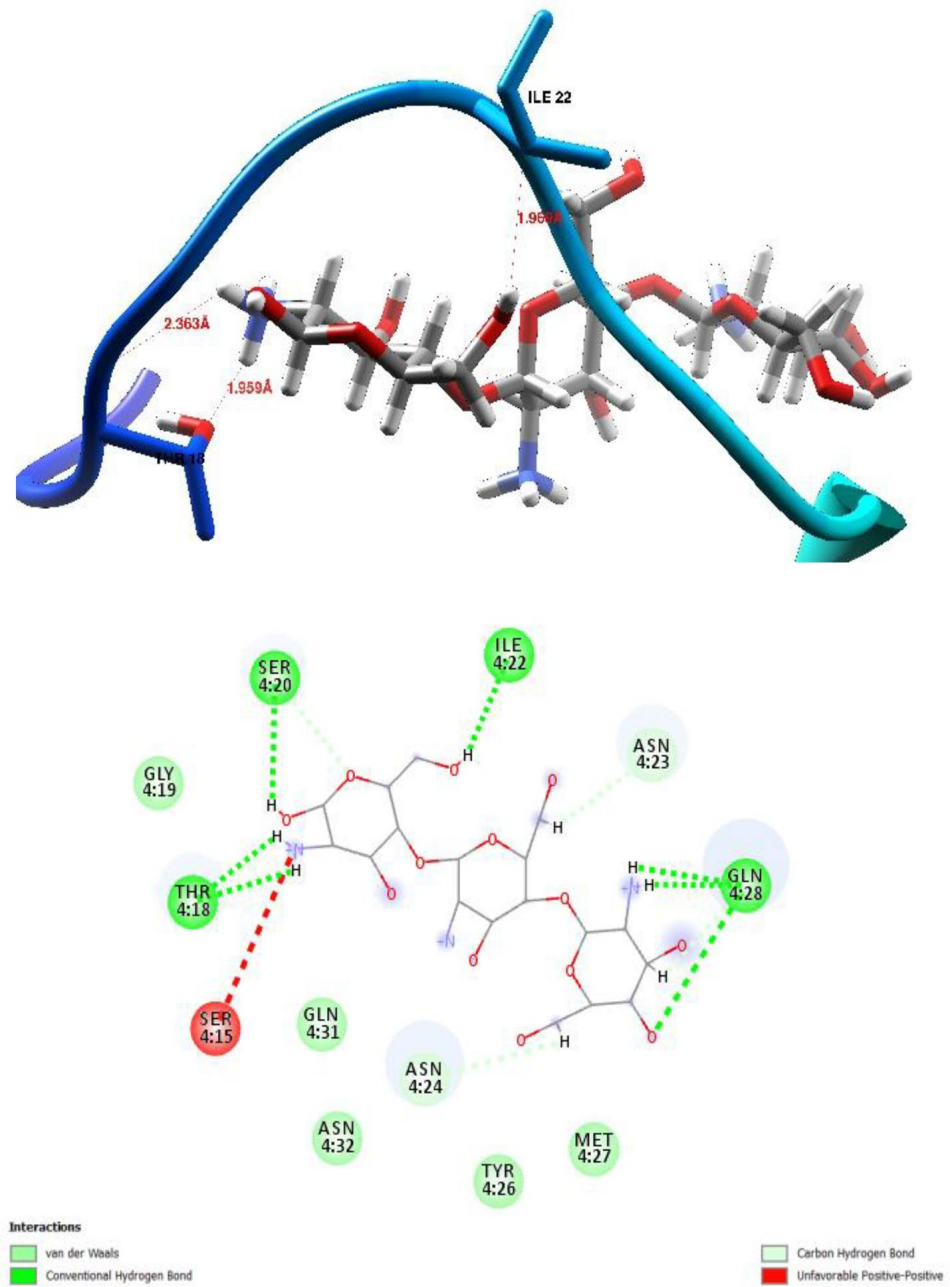
3D and 2D interaction of chitosan within the active sites of VP4 of FMDV capsid

The molecular docking as a computational analysis, executed to predict the binding modes and affinities of GA-NPs and Ch-NPs against six FMD viral proteins, represented in Table (2) & (3). According to binding affinities, GA-NPs gave the best results with RNA-dependent RNA polymerase (3D^pol^) followed by VP2, VP1, VP3, 3C protease (3C^pro^), then VP4. In contrast, Ch-NPs demonstrated superior findings with VP3 followed by 3D^pol^, 3C^pro^, VP2, VP1 then VP4. The 3D structure of viral proteins and 2D structure of ligands are shown in Fig. (4) and the 3D and 2D interactions of the nanoparticles with active sites of FMDV proteins were illustrated in (Figs. 5, 6, 7, 8, 9, 10, 11, 12, 13, 14, 15 and 16).

GA-NPs showed the strongest binding affinity toward RNA-dependent RNA polymerase (3D^pol^), with a binding energy of −8.974 kcal/mol through making eight hydrogen bonds with ASP X:245; HIS X:204; ARG X:193; ASP X:109; GLY X:299; ARG X:128; GLU X:112; ALA X:110 within the binding pocket, in addition to van der Waals and carbon-hydrogen interactions with different active site residues (Fig. 6; Table 2). In contrast, Ch-NPs revealed a binding affinity of −7.132 kcal/mol. They bonded to the active sites through 3 interactions, including conventional hydrogen bonds with SER X:304; ARG X:179; ILE X:180; VAL X:55; ALA X:243, carbon-hydrogen, and van der Waals interactions (Fig. 12; Table 3).

Conversely, Ch-NPs demonstrated greater binding affinity toward VP3 FMDV capsid (−7.173 kcal/mol) by forming eight conventional hydrogen bonds with ALA 3:171; TYR 3:169; SER 3:172; LYS 3:84; GLU 3:176; THR 3:177; THR 3:178; SER 3:80. Also, they establish two carbon-hydrogen bonds, several van der Waals interactions, and one salt bridge (Fig. 15; Table 3).

Compared with Ch-NPs, GA-NPs showed consistently higher binding affinities toward VP1, VP2, and VP3 FMDV capsid proteins, with docking scores of −8.450, −8.765, and − 8.424 kcal/mol, respectively (Table 2). The interaction between GA-NPs and VP1 FMDV was stabilized by 5 hydrogen bonds with THR 1:161; ASN 1:164; ARG 1:189; ARG 1:157; TYR 1:130, one carbon-hydrogen interaction, two alkyl interactions, several van der Waals interactions, and one unfavourable interaction (Fig. 7). Similarly, the compound binding to VP2 and VP3. Binding to VP2 showed five conventional hydrogen bonds with SER 2:28; THR 2:25; SER 2:24; ASN 2:202; GLU 2:108, one carbon-hydrogen interaction, hydrophobic (alkyl and pi-alkyl) interactions, and van der Waals interactions with multiple amino acid residues (Fig. 8), whereas binding to VP3 formed five hydrogen bonds with the following amino acids: GLY 3:129; TRP 3:183; ASP 3:78; HIS 3:85; GLN 3:76, together with van der Waals and carbon-hydrogen interactions (Fig. 9).

Among the analyzed viral proteins, VP4 displayed the weakest binding energies of −6.438 and − 4.887 kcal/mol for both GA-NPs and Ch-NPs, respectively. GA-NPs interacted with VP4 through formation of six hydrogen bonds with THR 4:18; GLN 4:28; ASN 4:32; SER 4:15; TYR 4:26; ASN 4:24, one carbon-hydrogen interaction, and van der Waals interactions (Fig. 10). In comparison, Ch-NPs formed four hydrogen bonds with THR 4:18; SER 4:20; ILE 4:22; GLN 4:28, van der Waals interactions, two carbon-hydrogen interactions, and one unfavourable positive-positive interaction (Fig. 16). Binding of these compounds to VP4 affects capsid integrity and RNA packaging.

GA-NPs docked with 3C protease (3C^pro^), exhibiting docking score of −6.732 kcal/mol (Table 2). The interaction stabilized by one hydrogen bond with HIS A:46, different van der Waals interactions, one carbon-hydrogen interaction, alkyl and Pi-Alkyl interactions, and unfavourable interactions (Fig. 5). Otherwise, Ch-NPs demonstrated a lower binding affinity (−5.796 kcal/mol) and established four hydrogen bonds with ILE A:30; ASP A:123; GLU A:50; HIS A:46, two carbon-hydrogen interactions, and van der Waals interactions (Fig. 11).

### In vitro cytotoxicity test

**Fig. 17:**
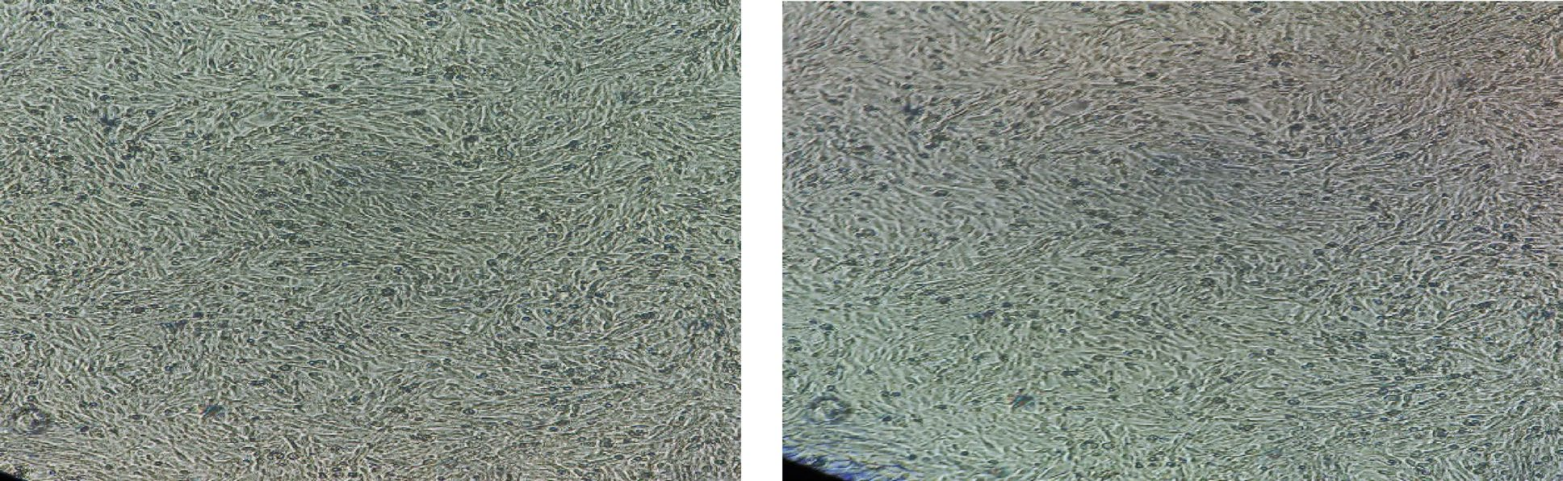
**a **and** b **demonstrate the in vitro cytotoxic effect of GA-NPs and Ch-NPs on BHK cells showing normal spindle shape with no deformity in cell shape (Untreated control BHK-21 cells, GA-NPs and Ch-NPs (2 mg/ml), GA-NPs and Ch-NPs (2x10^−1^ mg/ml), and GA-NPs and Ch-NPs (2x10^−2^ mg/ml)

The in vitro assay of GA-NPs and Ch-NPs cytotoxicity on BHK cell culture, using the dilutions 0 (undiluted); 1/10; and 1/100 (concentrations of 2; 2 × 10^− 1^; and 2 × 10^− 2^ mg nanoparticles/ml) manifested no cytotoxicity as tabulated in Table (4). These dilutions were entirely safe to BHK cells displaying normal growth behavior and normal cell shape as in Fig. (17).

**Table 9:** Virucidal assay of GA-NPs and Ch-NPs concentration against FMDV in G. pigs

**Tested dilution**	**Nanoparticles’ ** **Concentration ** **(mg/ml)**	**FMDV serotype**	** Virus sample** **Number**	**Number of experimental G. pigs**			**Virucidal action** **%**
				**Total number**	**No. of infected **	**No. of survived **	
**Undiluted**	2	O	V1	3	0	3	100
			V2	3	0	3	
			V3	3	0	3	
		A Iran 05	V4	3	0	3	100
			V5	3	0	3	
			V6	3	0	3	
		A Africa 2020	V7	3	0	3	100
			V8	3	0	3	
			V9	3	0	3	
		A Africa G IV Egypt 2022	V10	3	0	3	100
			V11	3	0	3	
			V12	3	0	3	
		A Venezuela	V13	3	0	3	100
			V14	3	0	3	
			V15	3	0	3	
		SAT2	V16	3	0	3	100
			V17	3	0	3	
			V18	3	0	3	
		Control	V19	3	3	0	-
**1/10**	2 x 10^-1^	O	V1	3	0	3	100
			V2	3	0	3	
			V3	3	0	3	
		A Iran 05	V4	3	0	3	100
			V5	3	0	3	
			V6	3	0	3	
		A Africa 2020	V7	3	0	3	100
			V8	3	0	3	
			V9	3	0	3	
		A Africa G IV Egypt 2022	V10	3	0	3	100
			V11	3	0	3	
			V12	3	0	3	
		A Venezuela	V13	3	0	3	100
			V14	3	0	3	
			V15	3	0	3	
		SAT2	V16	3	0	3	100
			V17	3	0	3	
			V18	3	0	3	
		Control	V19	3	3	0	-
**1/100**	2 x 10^-2^	O	V1	3	1	2	66.7
			V2	3	1	2	
			V3	3	1	2	
		A Iran 05	V4	3	1	2	44.4
			V5	3	2	1	
			V6	3	2	1	
		A Africa 2020	V7	3	1	2	66.7
			V8	3	1	2	
			V9	3	1	2	
		A Africa G IV Egypt 2022	V10	3	0	3	100
			V11	3	0	3	
			V12	3	0	3	
		A Venezuela	V13	3	1	2	77.8
			V14	3	1	2	
			V15	3	0	3	
		SAT2	V16	3	0	3	100
			V17	3	0	3	
			V18	3	0	3	
		Control	V19	3	3	0	-

### In vivo toxicity test

**Fig. 18:**
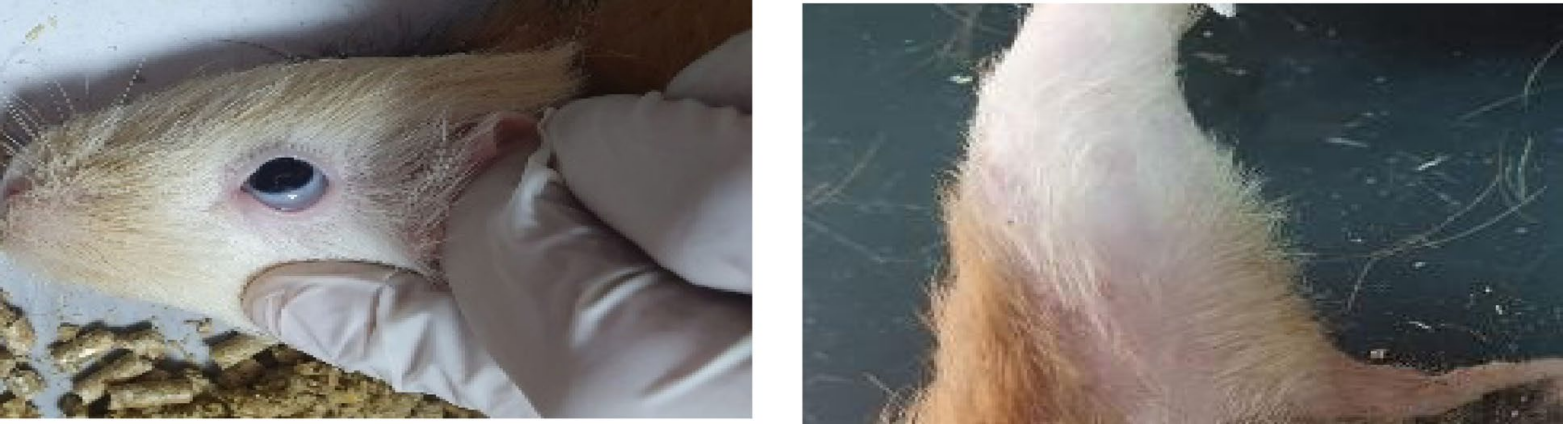
**a** and **b** demonstrate the in vivo toxicity of GA-NPs and Ch-NPs in G. pigs (no local reaction either in eye (fig-a) or at subcutaneous injection site (fig-b)) treated with GA-NPs and Ch-NPs (2 mg/ml), GA-NPs and Ch-NPs (2x10^−1^ mg/ml), and GA-NPs and Ch-NPs (2x10^−2^ mg/ml) as in untreated G. Pigs

The results of the in vivo assay of GA-NPs and Ch-NPs toxicity in G. pigs and mice as shown in Table (5) & (6) gave conformable results to the in vitro assay on BHK cells in various nanoparticles’ concentrations, where the 0.4 ml in G. pigs and 0.2 ml in mice of the following inoculated dilutions (0 (undiluted); 1/10; and 1/100 with concentrations of 2; 2 × 10^− 1^; and 2 × 10^− 2^ mg nanoparticles/ml) respectively exhibit no swelling or redness observed at the inoculation site as in Fig. (18). The in vivo toxicity assessment was limited to the observation of local inoculation site reactions.

### In vitro antiviral assay

**Fig. 19:**
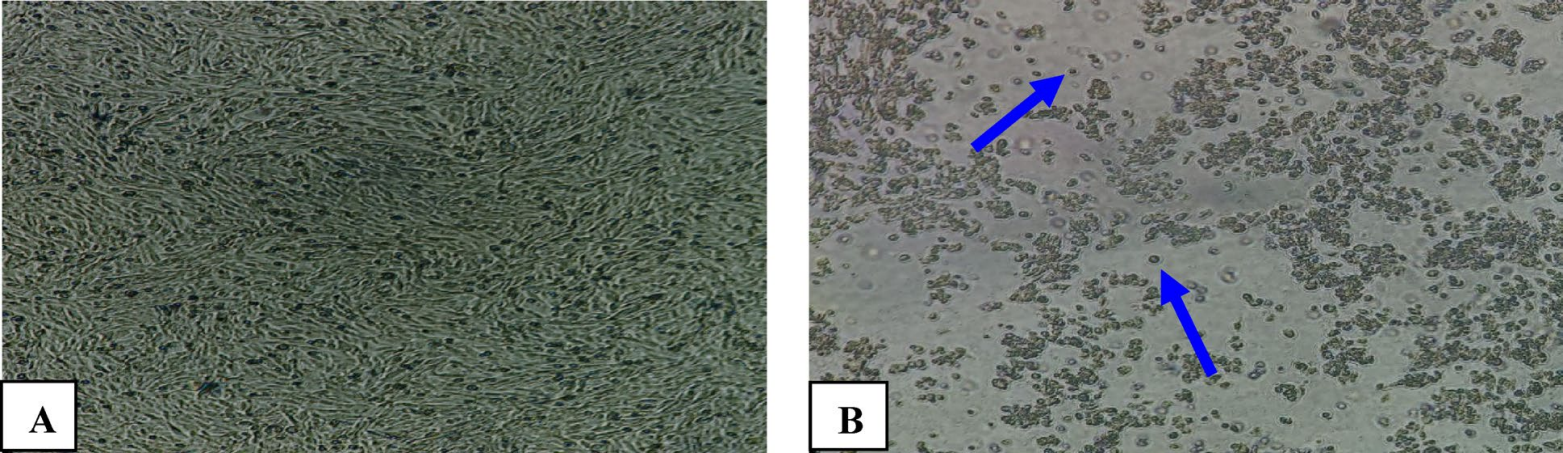
Virucidal assay of GA-NPs and Ch-NPs concentration against FMDV on BHK cells. **A:** Normal spindle cell shape (control cells and GA-NPs and Ch-NPs treated cells) with virucidal action **B:** CPE induced by FMD viruses showing cell rounding revealing no virucidal action

The concentrations (2 and 2 × 10^− 1^ mg nanoparticles/ml) of nanoparticles on BHK cell culture showed complete antiviral effect (100% decline in the virus titer) against O PanAsia-2, A Iran 05, A Venezuela, A Africa 2020, A Africa G IV Egypt 2022, and SAT2/EGY/2012 FMDV serotypes except the dilution 1/100 (2 × 10^− 2^ mg nanoparticles/ml) which revealed entire antiviral effect against A Iran 05 and A Africa 2020, whereas presented antiviral influence of 81.25%; 92.86%; 80%; and 92.31% against serotype O; A Africa G IV Egypt 2022; A Venezuela; and SAT2 respectively as mentioned in Table (7). The untreated cells showed characteristic CPE as shown in Fig. (19).

### In vivo antiviral assay

#### In baby mice

**Fig. 20:**
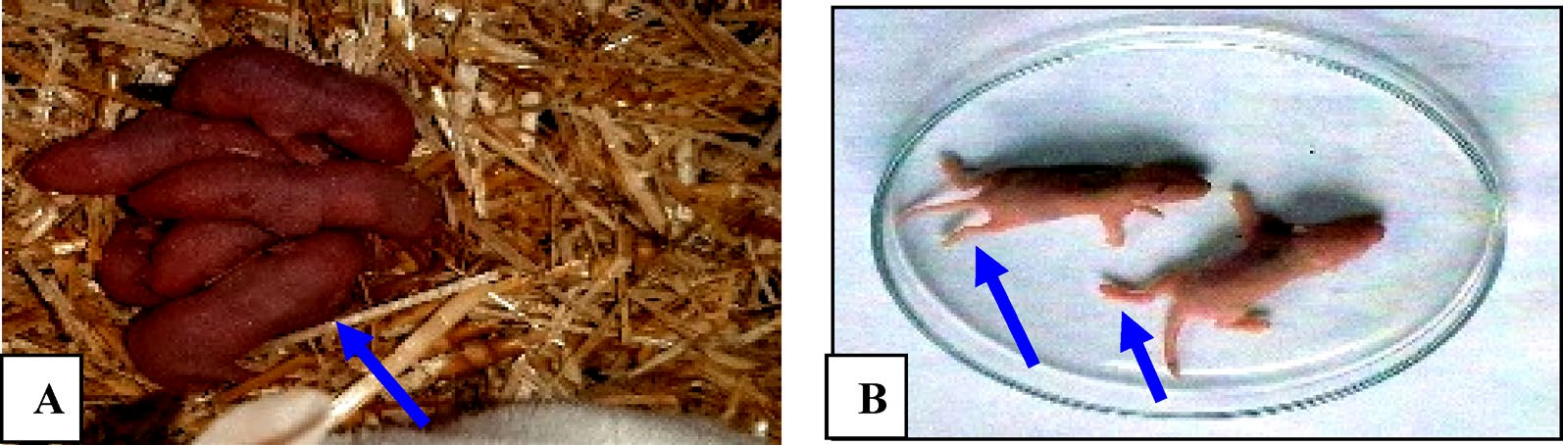
The virucidal assay of GA-NPs and Ch-NPs against FMDV in mice. **A: **Baby normal control mice and GA-NPs and Ch-NPs treated mice with virucidal action **B:** Mice showing paralysis in hind limbs without virucidal action

The virucidal effect of GA-NPs and Ch-NPs executed in mice experimentally infected with O PanAsia-2, A Iran 05, A Venezuela, A Africa 2020, A Africa G IV Egypt 2022, and SAT2/EGY/2012 FMDV serotypes, revealed that the determined concentrations (2; 2 × 10^− 1^; and 2 × 10^− 2^ mg nanoparticles/ml) endured the experimental infection with a percentage of 100 up to a concentration of 2 × 10^− 1^ mg/ml against FMDV serotypes (Table 8). While 2 × 10^− 2^ mg nanoparticles/ml showed 66.7%; 44.4%; 66.7%; and 77.8% protection against serotype O; A Iran 05; A Africa 2020; and A Venezuela, respectively, but 100% protection against serotype A Africa G IV Egypt 2022 and SAT2. The untreated infected mice manifested characteristic paralysis of the hind limbs as displayed in Fig. (20).

#### In G. pigs

**Fig. 21:**
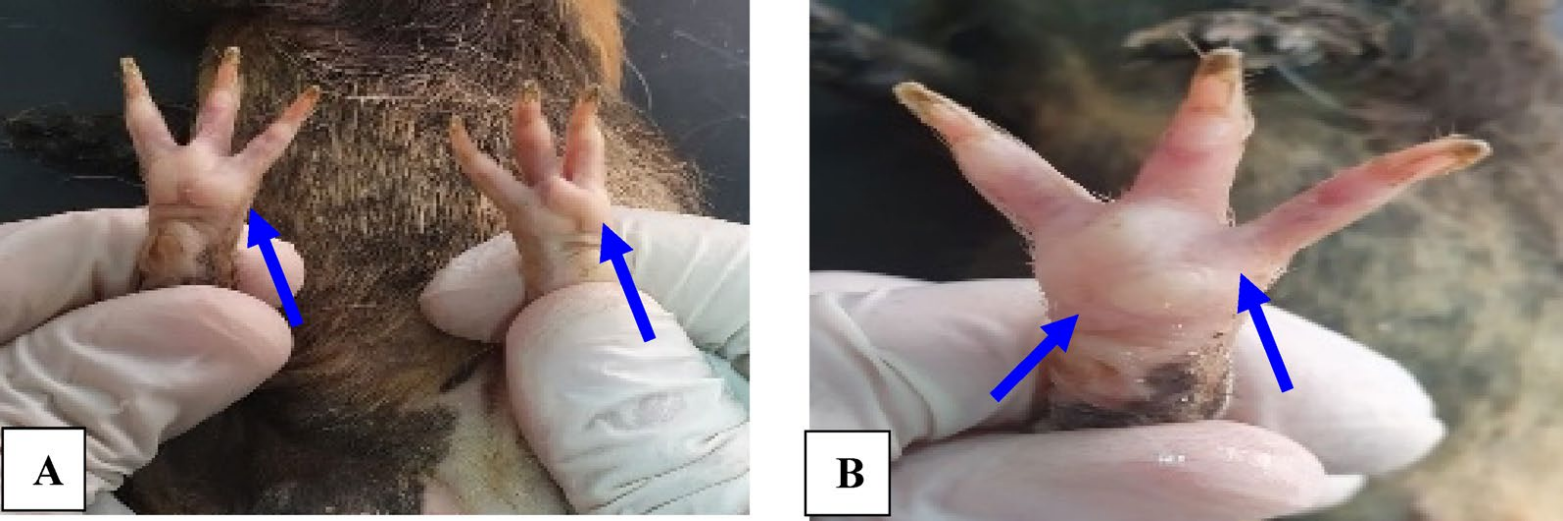
The virucidal effect of GA-NPs and Ch-NPs against FMDV in G. pigs. **A:** Normal planter surface of limb without vesicles (untreated control G. pigs and GA-NPs and Ch-NPs treated G. pigs) with virucidal action **B:** Vesicle in planter surface of limb without virucidal action

The virucidal effect of GA-NPs and Ch-NPs applied in G. pigs experimentally infected with O PanAsia-2, A Iran 05, A Venezuela, A Africa 2020, A Africa G IV Egypt 2022, and SAT2/EGY/2012 FMDV serotypes, revealed that the determined concentrations (2; 2 × 10^− 1^; and 2 × 10^− 2^ mg nanoparticles/ml) endured the experimental infection at percentage of 100 up to a concentration of 2 × 10^− 1^ mg/ml. While 2 × 10^− 2^ mg nanoparticles/ml showed 66.7%; 44.4%; 66.7%; and 77.8% protection against serotype O; A Iran 05; A Africa 2020; and A Venezuela, respectively, but 100% protection against serotype A Africa G IV Egypt 2022 and SAT2 (Table 9). The untreated infected G. pigs showed discriminatory vesicle on planter surface of limbs and other symptoms as in Fig. (21).

## Discussion

The nanotechnology was integrated with small molecule-structured nanomaterials in order to reduce the adverse effects of natural compounds, upgrade the compound efficiency and targeted drug delivery. These nanoparticles have distinctive traits of reduced toxicity, water solubility, cost-effective synthesis, and biocompatibility [24].

Some studies have been revealed that using nanoparticles is considered as an alternative form of healing to treat various diseases such as FMD. Nano formulations are incrementally being carried out for clinical translation and are being considered a new framework against infectious diseases. Natural polymers like chitosan and glycyrrhizic acid are favored to design nanoparticles due to their biocompatibility, biodegradation, and long shelf-life [25].

This research was designed to evaluate the influence of GA-NPs and Ch-NPs, as potential natural antiviral agents, constraining FMD outbreaks. It was supported computationally using molecular docking analysis.

The surface properties of GA-NPs and Ch-NPs were characterized by TEM examination (Figs. 1 and 2). They were spherical particles in the range of 20–80 nm for GA-NPs and 10–40 nm with some particles reaching up to 70 nm for Ch-NPs, with uniform distribution and minor clustering, attributable to drying throughout sample preparation and polymer–polymer interactions. The average zeta potential of GA-NPs possessed a negative surface charge of −14.25 mV due to carboxyl groups, with average particle size of 72.22 nm. These findings align with the study previously reported by [24], where GA-NPs average particle size of 70.65 nm and average zeta potential with negative surface charge of −32.7 mV. Whereas Ch-NPs, in the present study, have positive surface charge of 7.3 mV due to –NH₃⁺ group, with average particle size of 40.69 nm.

Furthermore, the FTIR spectra of both GA-NPs and Ch-NPs demonstrated distinctive peak shifts and band broadening, particularly in the O–H/O–H–N stretching regions, indicating enhanced hydrogen bonding and intermolecular interactions within the nanoparticle matrix. In GA-NPs, changes in the carbonyl and C–O/C–O–C bands suggest the incorporation of carboxyl and glucuronic acid moieties in nanoparticle stabilization, while in Ch-NPs, shifts in the amino- and carbonyl-related bands indicate the participation of chitosan functional groups in the interaction process. The absence of new absorption bands in both formulations indicates that nanoparticle formation occurred through non-covalent (physical) interactions without altering the chemical structures of glycyrrhizic acid or chitosan, confirming the successful encapsulation of glycyrrhizic acid.

The computational analysis utilized molecular docking carried out to estimate binding modes and affinities of GA-NPs (PubChem CID: 14982) and Ch-NPs (PubChem CID: 129662530) against six FMDV proteins: 3C protease (3C^pro^) (PDB ID: 2WV4); RNA-dependent RNA polymerase (3D^pol^) (PDB ID: 2E9R); and FMDV complex (VP1, VP2, VP3, and VP4) (PDB ID: 7DSS). The two-dimensional and three-dimensional interactions among nanoparticles as ligands and protein active sites as receptors, including van der Waals, carbon-hydrogen, hydrogen bonds, salt bridge, and alkyl and Pi-alkyl, are included in Fig. (5–16).

Each target protein has a substantial function in FMDV. 3C^pro^ is a chymotrypsin like cysteine protease contributes to most of the viral polypeptide cleavage [26]. FMDV 3C^pro^ cleavage sites exhibit significant heterogeneity, with cleavage happening among multiple dipeptides, including Gln-Gly, Glu-Gly, Gln-Leu, and Glu-Ser [27]. It’s also responsible for suppression of host cell transcription [28]. 3D^pol^ is a highly conserved [29] viral encoded RNA dependent RNA polymerase (RdRp) [30]. It’s the catalytic component of RNA replication to synthesize positive and negative sense genome and participates in the RNA viruses’ life cycle [31]. Its structure looks like cupped ‘right hand’ composed of ″palm‶, ″fingers‶ and ″thumb″ subdomains. The palm domain is involved in structural integrity, nucleotide recognition and binding, phosphoryl transfer, and priming nucleotide binding [32], and includes a conserved YGDD sequence and a highly conserved D residue as active sites [33].

Additionally, the remaining 4 capsid proteins known as VP1, VP2, VP3, VP4 are integrated to form the viral icosahedral structure [34]. VP1 protein associated with formation of neutralizing antibodies and attachment to susceptible cells [35]. VP1 contains G-H loop (with RGD motif) that is responsible for binding to integrin receptors on host cells allowing viral entry [36, 37]. Also, it interacts with VP2 and VP3 to assemble and stabilize viral capsid. VP2 protein involved in virion structural stability and maturation [38]. VP3 protein responsible for capsid stability [39]. VP4 is an internal capsid protein helps to maintain the integrity of the viral particle, interacting with viral RNA assisting in its encapsulation inside the capsid, and helps in viral uncoating during infection [40].

The greater binding affinity of GA-NPs, together with the higher number of conventional hydrogen bonds, suggests a more stable interaction with the 3D^pol^ active site than Ch-NPs. Because 3D^pol^ is important for viral RNA synthesis, these interactions may interfere with polymerase function and contribute to the antiviral activity observed in vitro. Also, Binding of ligands to the active pocket may impede nucleotide addition and RNA elongation and synthesis, thus preventing viral genome proliferation. These results are consistent with [41, 42], who investigate the RdRp structure of FMDV in complex with a template-primer RNA Deca-nucleotide at 3 Å resolution and in a free form at 1.9 Å resolution, as the interactions involved amino acid residues correlated with conserved catalytic motifs contributes to viral RNA synthesis.

In addition to targeting viral replication, the consistently higher binding affinities of GA-NPs toward the FMDV capsid proteins suggest that glycyrrhizic acid may interact more stably with structural proteins involved in viral attachment and capsid integrity. In particular, the interaction with VP1 is of interest because this protein contains the G–H loop, which mediates binding to integrin receptors on host cells. All these results were supported by [43], who mentioned that binding of two potent protective neutralizing antibodies (M8 and M170) with residues located in G-H loop region of VP1 FMDV, which is responsible for integrin receptor binding, can make complete inhibition of the viral adhesion to the host cell receptors, obstructing host cell recognition, and preventing viral entry and infectivity. Moreover, interactions occurred during docking analysis against VP2 and VP3 may change capsid structure or antigenic regions, possibly influence viral infectivity and capsid stability, and hinder virion formation.

Besides targeting structural proteins and the viral polymerase, both GA-NPs and Ch-NPs interactions with 3C^pro^ suggests a potential mechanism underlying their antiviral activity. The FMDV 3C^pro^ is essential for cleavage of the viral polyprotein into functional proteins required for viral replication. Therefore, ligand binding within or near the catalytic pocket may interfere with protease activity, impair polyprotein processing, and consequently reduce viral replication. This interpretation come in parallel with [44], who reported that inhibition of FMDV 3C^pro^ disrupts viral polyprotein cleavage and suppresses viral multiplication.

The in vitro assay of GA-NPs and Ch-NPs cytotoxicity on BHK cell culture, using the microtiter technique, indicated normal growth behavior with normal cell shape at concentrations 2 to 2 × 10^− 2^ mg/ml (Fig. 17). These findings come parallel with those of [45] who confirmed that the polymeric nanoparticles - glycyrrhizic acid (PNPS-GA) have safety range of 50 µl/ml with no significant cytotoxic effects or morphological changes on MARC-145 cell line, suggesting that these nanoparticles are regarded as biocompatible nanocarriers for drug delivery.

With regard to the in vivo assay of GA-NPs and Ch-NPs toxicity in baby Swiss mice and G. pigs, it yielded consistent results to the in vitro assay on BHK cells when inoculated with divergent dilutions (0 (undiluted); 1/10; and 1/100 with concentrations of 2; 2 × 10^− 1^; and 2 × 10^− 2^ mg nanoparticles/ml). Both of them didn’t display any clinical signs (Fig. 18) [46]. affirmed these outcomes by combining glycyrrhizic acid nanoparticles into micelles with curcumin to investigate their potentiality to avoid hearing loss. The micelles administered orally with tympanic injection (40 mg/kg) in guinea pig models. Under experimental conditions, GA nano-formulations manifested no notable systemic toxicity.

Concerning the in vitro antiviral impact of GA-NPs and Ch-NPs on FMDV serotypes (O Pan-Asia/2012; A Iran 05; A Africa 2020; A Africa G IV Egypt 2022; A Venezuela; and SAT2/EGY/2012), the present study revealed that the safe concentrations (2 and 2 × 10^− 1^ mg nanoparticles/ml) of GA-NPs and Ch-NPs on BHK cell culture elicited entire antiviral effect (100% decrease in the virus titer) against the six FMD virus serotypes except the dilution 1/100 (2 × 10^− 2^ mg nanoparticles/ml) which revealed complete antiviral effect against A Iran 05 and A Africa 2020 only, while didn’t consider as antivirus against serotype O; A Africa G IV Egypt 2022; A Venezuela; and SAT2. The untreated cells presented characteristic CPE. Such antiviral action coincides with the findings of [24]. GA-NPs could inhibit the propagation of MHV-A59 and diminish expression of mRNA at 12, 24, 36, and 48 h post infection (HPI). In the cell supernatant, GA-NPs limited the viral titers by a maximum of 10^5^ times. Also, it was found that the suppressive effect of GA-NPs is a dose-related, and the inhibition effect attained 80% when the concentration of GA-NPs was 0.4 mg/ml.

Otherwise, fabricated chitosan-coated silver nanoparticles exhibited a good in vitro biocompatibility on Vero-E6 cells against SARS-CoV-2 as stated by [47]. Compared to AgNPs and Ch-NPs alone, Ch/AgNPs validated greater antiviral efficiency with the maximum selectivity index (SI = 33.53). It suppressed RdRp activity (IC_50_ = 32.67 µg/ml), Cathepsin L (CTSL) (IC_50_ = 13.83 µg/ml), and SARS-CoV-2 spike/ACE2 binding (IC_50_ = 303.3 µg/ml).

The results of the in vivo study of GA-NPs and Ch-NPs antiviral activity in G. pigs and mice experimentally infected with the six FMDV serotypes, indicated that the measured concentrations (2; 2 × 10^− 1^; and 2 × 10^− 2^ mg nanoparticles/ml) resisted the experimental infection at percentage of 100 up to a concentration of 2 × 10^− 2^ mg/ml. While 2 × 10^− 2^ mg nanoparticles/ml didn’t regard as antivirus against serotype O; A Iran 05; A Africa 2020; and A Venezuela, but provide 100% protection against serotype A Africa G IV Egypt 2022 and SAT2 (Tables 8 and 9). The untreated infected G. pigs manifested discriminatory vesicle on planter surface of limbs and other symptoms, in contrast, the untreated infected mice displayed paralysis of the hind limbs. These results reflect the antiviral activity of GA-NPs as described by [24]. When female BALB/c mice inoculated, by nasal administration, with the murine coronavirus mouse hepatitis virus strain A59 (MHV-A59), the viral load decreased in lung and liver tissues, alleviating organ and tissue damage. Compared with untreated infected controls, GA-NPs rendered a considerable increased survival rate to infected mice.

In contrast [47], monitored the virucidal potential of Ch-NPs by proving that chitosan coating decreased the tissue toxicity enhanced by AgNPs in liver, kidney, and lung tissues, using histopathological studies on albino rat models. Also, the present results showed the same expectations of [48] who studied the antiviral activity of chitosan nanoparticles (CNPs) and curcumin-loaded chitosan nanoparticles (Cur-CNPs) against two virulent Newcastle Disease Virus (NDV) strains, B3/MIB/PKD/23 and L2/MIB/PKD/23, isolated from poultry. The greatest protection, measured using MTT assay, with Cur-CNPs (0.75:1 ratio), attaining 44.1% and 47.1% protection for 2 strains. Relative to CNPs, quantitative real-time PCR verified consequential viral load decrements (*p* < 0.001) with Cur-CNPs, also, viral titer suppression assays showed a decline of 3 log_10_ TCID_50_ and a 2-fold diminution in hemagglutination titers. These observations support that nanoparticle-based strategies may represent prospective approaches for managing FMD viral infection in vulnerable animal models.

Although the present findings support the antiviral potential of GA-NPs and Ch-NPs against multiple FMDV serotypes, the antiviral evaluation was conducted as a single biological experiment with technical replicate wells rather than independent biological replicates. Consequently, statistical analysis of the antiviral efficacy was not applicable, and the observed antiviral effects should be interpreted as preliminary. Future studies should validate these findings using independent biological replicates and more comprehensive experimental designs.

## Conclusion

Based on the present study findings, GA-NPs and Ch-NPs suggested antiviral activity against various FMDV serotypes in computational, in vitro, and in vivo evaluation. Computational analysis suggested favorable interactions with viral targets, while in vitro and in vivo assessment supported their potential as natural antiviral candidates under the experimental conditions. Conversely, the findings should be interpreted in light of the study limitations, including the restricted evaluation of systemic in vivo toxicity. Further studies incorporating comprehensive toxicity evaluation, conformation of antiviral activity using independent biological replicates, and efficacy testing in target livestock species are needed to confirm these preliminary findings and determine their potential application in FMD control. Notably, more studies in cattle and sheep, the principal natural hosts of FMDV, are recommended to evaluate the antiviral efficacy, safety, optimal dosage, and therapeutic potential of these nanoparticle formulations during natural FMDV infection. In addition, we recommend future investigations to evaluate whether these nanoparticle formulations exhibit antiviral activity against other economically important veterinary viruses, such as Rift Valley fever virus, which causes substantial economic losses in livestock production in Egypt.

## Data Availability

No datasets were generated or analysed during the current study.

## Abbreviations

FMD: Foot and Mouth Disease
FMDV: Foot and Mouth Disease Virus
SAT1: South African Territories 1
SAT2: South African Territories 2
SAT3: South African Territories 3
GA-NPs: Glycyrrhizic Acid Nanoparticles
Ch-NPs: Chitosan Nanoparticles
Nm: nanometer
GA: Glycyrrhizic Acid
Ch: Chitosan
VSVRI: Veterinary Serum and Vaccine Research Institute
SDF: Structure Data File
RCSB PDB: Research Collaboratory for Structural Bioinformatics Protein Data Bank
VP1: Viral Protein 1
VP2: Viral Protein 2
VP3: Viral Protein 3
VP4: Viral Protein 4
FMDRD: Foot and Mouth Disease Research Department
TPP: Tripolyphosphate
DLS: Dynamic Light Scattering
TEM: Transmission Electron Microscope
FT-IR: Fourier Transform Infrared Spectroscopy
BHK_21_: Baby Hamster Kidney-21 cells
MEM: Minimum Essential Medium
I/P: Intraperitonially
CPE: Cytopathic Effect
TCID_50_: Median Tissue Culture Infectious Dose
ID_50_: Median Infectious Dose
3D^pol^: RNA-dependent RNA polymerase
3C^pro^: 3C protease
RdRp: RNA-dependent RNA polymerase
PNPS-GA: Polymeric Nanoparticles - Glycyrrhizic Acid
MARC-145: Monkey kidney epithelial cell line derived from the African green monkey (*Cercopithecus aethiops*)
HPI: Hour Post Infection
BALB/c mice: Bagg Albino mice
MHV-A59: Murine Hepatitis Virus strain A59
SARS-CoV-2: Severe Acute Respiratory Syndrome Corona Virus-2
SI: Selectivity Index
AgNPs: Silver Nanoparticles
IC_50_: Half-maximal Inhibitory Concentration
CTSL: Cathepsin L
ACE2: Angiotensin-Converting Enzyme 2
NDV: Newcastle Disease Virus
Cur-CNPs: Curcumin-loaded Chitosan Nanoparticles
CNPs: Chitosan Nanoparticles
MTT: 3-(4,5-dimethylthiazol-2-yl)-2,5-diphenyl-2 H-tetrazolium bromide
